# Supplementary material for: Genetic and epigenetic basis of hepatoblastoma diversity
Source: Nat Commun. 2021 Sep 20;12:5423. doi: 10.1038/s41467-021-25430-9 (PMC8450290; doi:10.1038/s41467-021-25430-9)
Supplement: Supplementary file 1 — Supplementary information [file 41467_2021_25430_MOESM1_ESM.pdf]

Genetic and epigenetic basis of hepatoblastoma diversity

Genta Nagae, et al.

Supplementary Table 1. Summary of clinical and pathological features of 163 childhood liver cancer cohorts.

**Gender**			F1	31	19.1%
Male	93	57.1%	E1-2	6	3.7%
Female	70	42.9%	N1	1	0.6%
**Birth weight (g)**			H1	19	11.7%
Non-VLBW (>1500)	120	73.6%	M1	32	19.6%
VLBW (<1500)	26	16.0%			
unknown	17	10.4%	**Serum AFP level (ng/mL)**		
**Age at diagnosis**			>1000	153	93.9%
< 2 year old (infant)	101	62.0%	100-999	9	5.5%
2-7 year old (child)	47	28.8%	<100	1	0.6%
≥ 8 year old (tween)	15	9.2%			
			**Pathological subtypes***		
**PRETEXT**			Mixed epithelial	83	51.0%
I	19	11.7%	Fetal	27	16.6%
II	47	28.8%	（Fetal, mitotically active	3	1.8%）
III	58	35.6%	Embryonal	13	8.0%
IV	39	23.9%	MEM	20	12.3%
**Annotation factors**			(MEM with teratoid)	5	3.1%）
None	100	61.3%	Macrotrabecular	5	3.1%
P1-2	13	8.0%	TLCT (HCN-NOS)	6	3.7%
V1-3	11	6.7%	HCC	9	5.5%

PRETEXT, pretreatment extent of disease; P, portal vein involvement; V, involvement of hepatic veins, the vena cava or both; F, tumor multifocality; E, extrahepatic abdominal disease; N, lymph node metastases; H, rupture or intraperitoneal hemorrhage; M, distant metastases; AFP, alpha-fetoprotein; TLCT, transitional cell tumor of the Liver; HCN-NOS, hepatocellular malignant neoplasm-not otherwise specified; HCC, hepatocellular carcinoma.

* Pathological subtypes were reclassified according to the current international classification[1].

Supplementary Table 2. Pathogenic germline mutations in the cancer-predisposing genes in 147 Japanese patients with HBL.

Age(yrs)	Birth weight*	PRE TEXT	Gene	Nucleotide change	Protein change	Significance in ClinVar (March 5, 2019)	*CTNNB1* status	Family history of cancer
1	NBW	II	*APC*	NM_000038:c.C646T	p.R216X	Pathogenic	WT	Familial adenomatous polyposis
1	LBW	II	*APC*	NM_000038:c.C1495T	p.R499X	Pathogenic	WT	Familial adenomatous polyposis
1	NBW	II	*APC*	NM_000038:c.3221delC	p.T1074fs	.	WT	None
1	NBW	IV	*APC*	NM_000038:c.3646_3653del	p.E1216fs	.	WT	None
2	NBW	I	*APC*<br>*ATM*	NM_000038:c.2805delC<br>NM_000051:c.7878_7882del	p.Y935X<br>p.A2626fs	Pathogenic<br>.	WT	None
1	NBW	IV	*BRCA1*	NM_007294:c.2389_2390del	p.E797fs	Pathogenic	Somatic mutation	None
1	NBW	IV	*BRCA2*	NM_000059:c.C9382T	p.R3128X	Pathogenic	WT	None
1	NBW	IV	*PMS2*	NM_000535:c.538-2A>G	.	Pathogenic/ Likely pathogenic	Somatic deletion	None
3	NBW	IV	*TP53*	NM_000546:c.G473A	p.R158H	Pathogenic/ Likely pathogenic	WT	None

*LBW, low birth weight, < 2500g; NBW, normal birth weight, ≥ 2500g.

None of the variants was found in Japanese allele frequency database (HGVD v2 and ToMMo 2K).

The following 40 genes were analyzed for germline mutations: *APC, ATM, AXIN1, AXIN2, BAP1, BARD1, BMPR1A, BRCA1, BRCA2, BRIP1, CDH1, CDKN2A, CHEK2, CTNNB1, EGFR, EPCAM, FANCM, FH, HNF1A, MLH1, MSH2, MSH6, MUTYH, NBN, NF1, PALB2, PMS2, POLD1, POLE, PTEN, RAD50, RAD51C, RAD51D, RNF43, SMAD4, STK11, TP53, TSC1, TSC2, VHL*. Most of these genes were selected due to them being well-established hereditary cancer genes. We supplemented them with the regulators of Wnt/β-catenin signaling (*CTNNB1, AXIN1, AXIN2, RNF43*) and somatic driver genes of adult liver cancer (*HNF1A, TSC1, TSC2)*

Supplementary Fig. 1

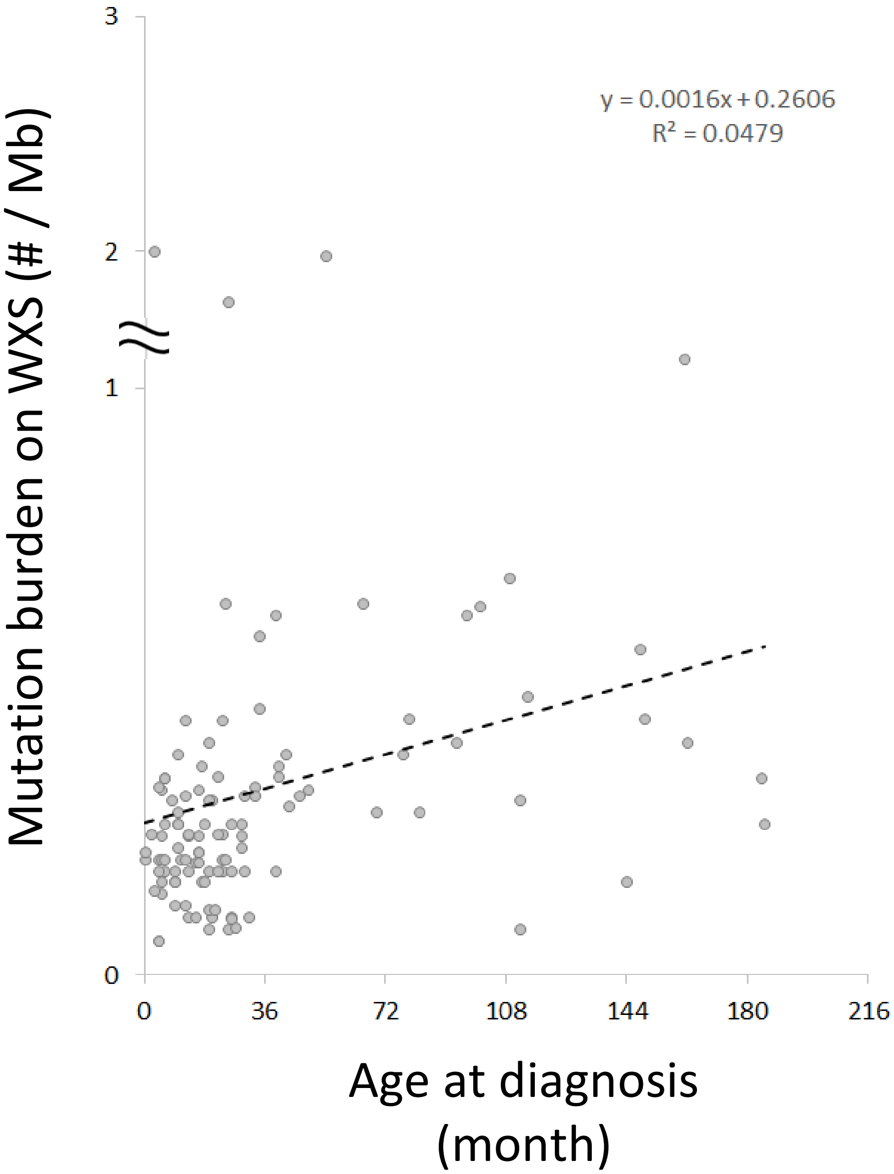

Supplementary Fig. 1: Correlations between the age at diagnosis and the number of somatic mutations detected by whole-exome sequencing (WXS) across 112 childhood hepatoblastoma (HB).

Supplementary Fig. 2

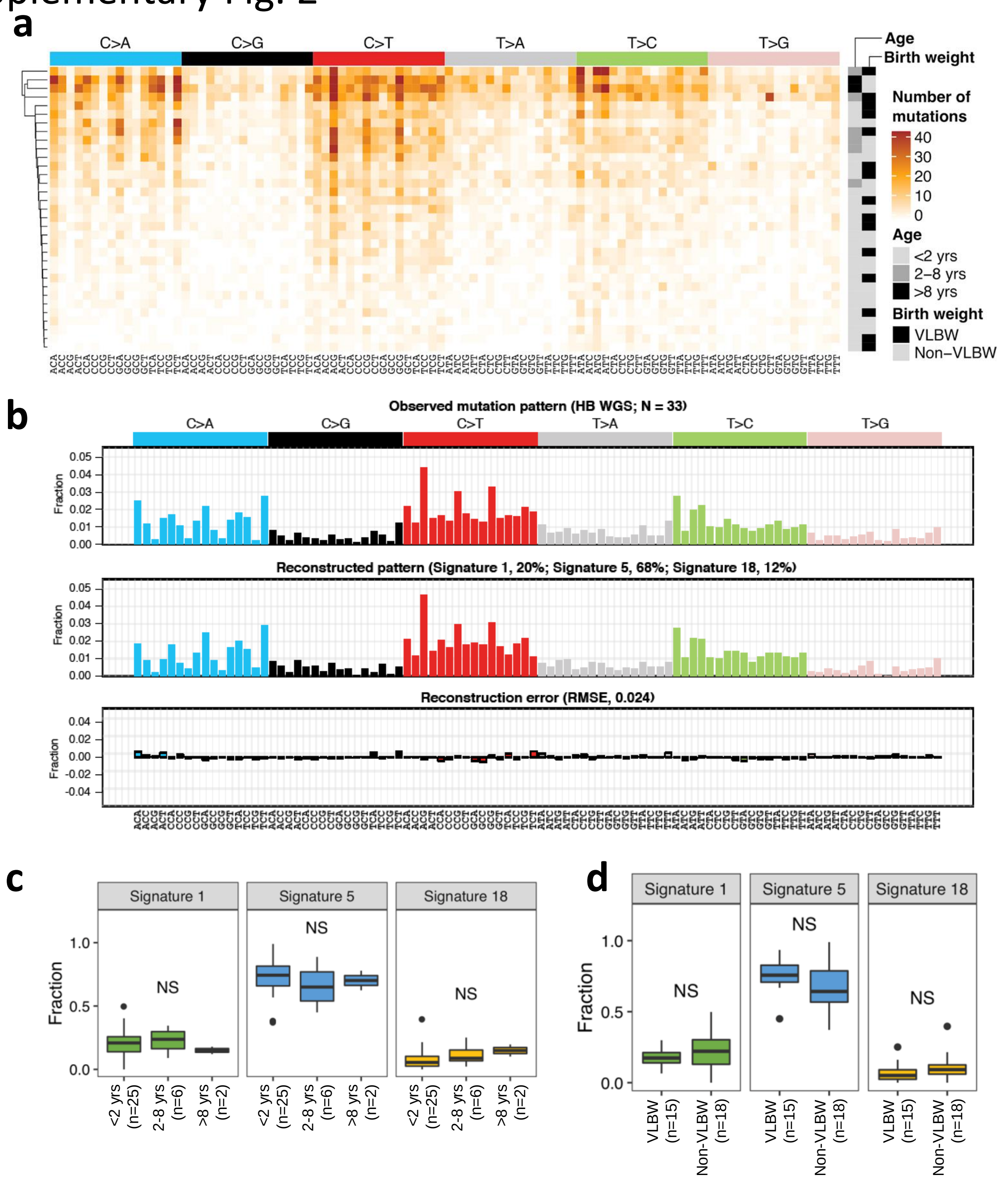

Supplementary Fig. 2: Mutational signatures in whole-genome sequencing (WGS) of 33 childhood hepatoblastoma (HB) genomes.
a, The heat map of mutational patterns on WGS (horizontal axis) across the 33 HB genomes (vertical axis). **b**, Observed and reconstructed trinucleotide substitution patterns. Single nucleotide variants (SNVs) in the 33 HB WGS data were pooled and decomposed into Signature 1, Signature 5, and Signature 18 of COSMIC. RMSE, root mean squared error. **c-d**, Box plot represents the fraction of Signature 1, 5, and 18 by age at diagnosis (c, *p*-values > 0.05 by Kruskal-Wallis test) and birth weight (d, *p*-values > 0.05 by Wilcoxon rank-sum test). The center lines show the medians, the tops and bottoms of boxes show quartiles, and the whiskers show the extremes within the range of the medians ± 1.5 × the interquartile ranges.

Supplementary Fig. 3

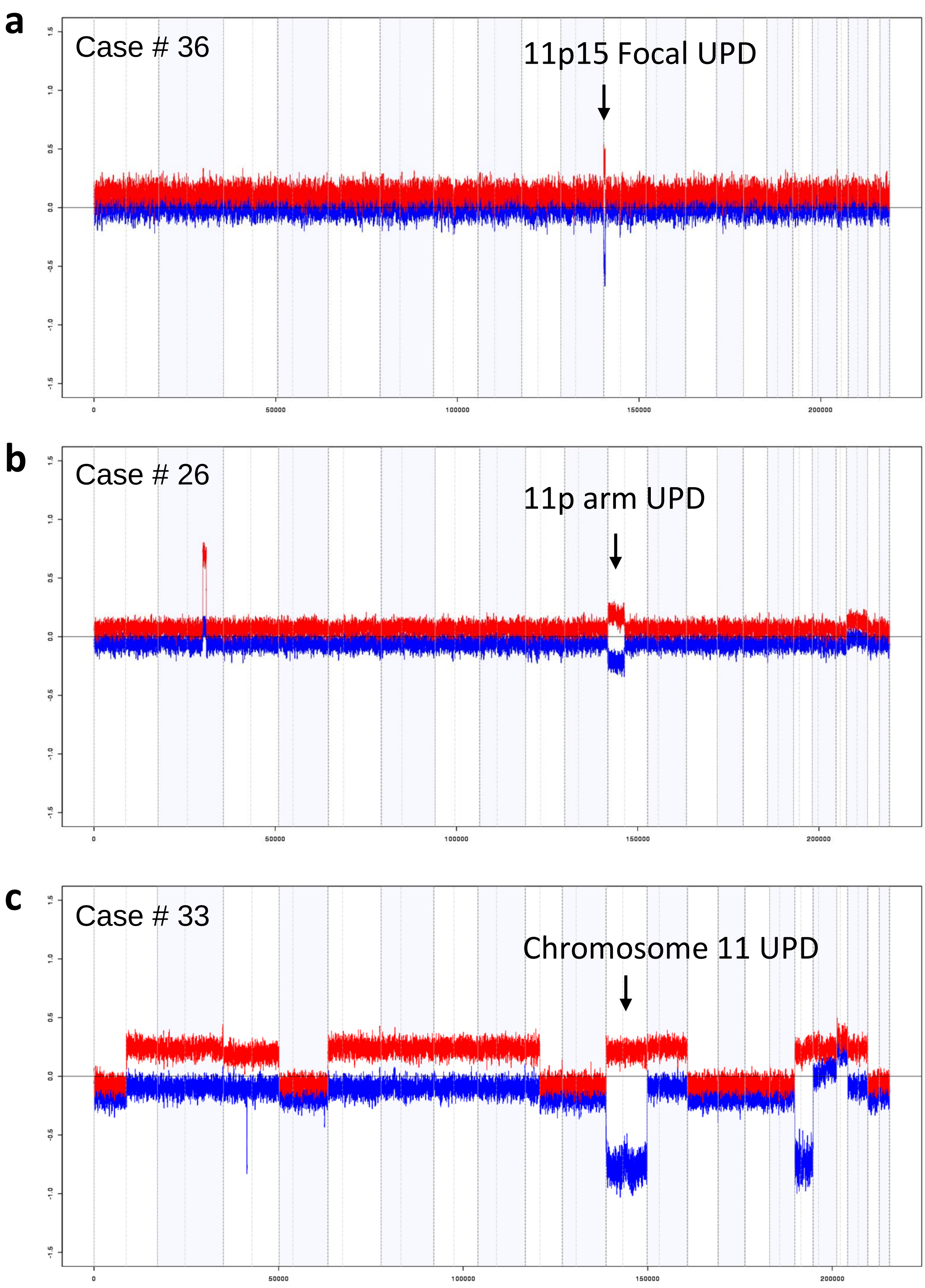

Supplementary Fig. 3: Copy number analysis of chromosome 11 by high-density SNP arrays.
Allele frequencies were quantitated by GEMCA and plotted as A allele (red) and B allele (blue). Three representative uniparental disomy (UPD) cases are shown: **a**, Chr.11p15 focal UPD; **b**, Chr.11p arm-wide UPD; **c**, chromosome 11-wide UPD.

Supplementary Fig. 4

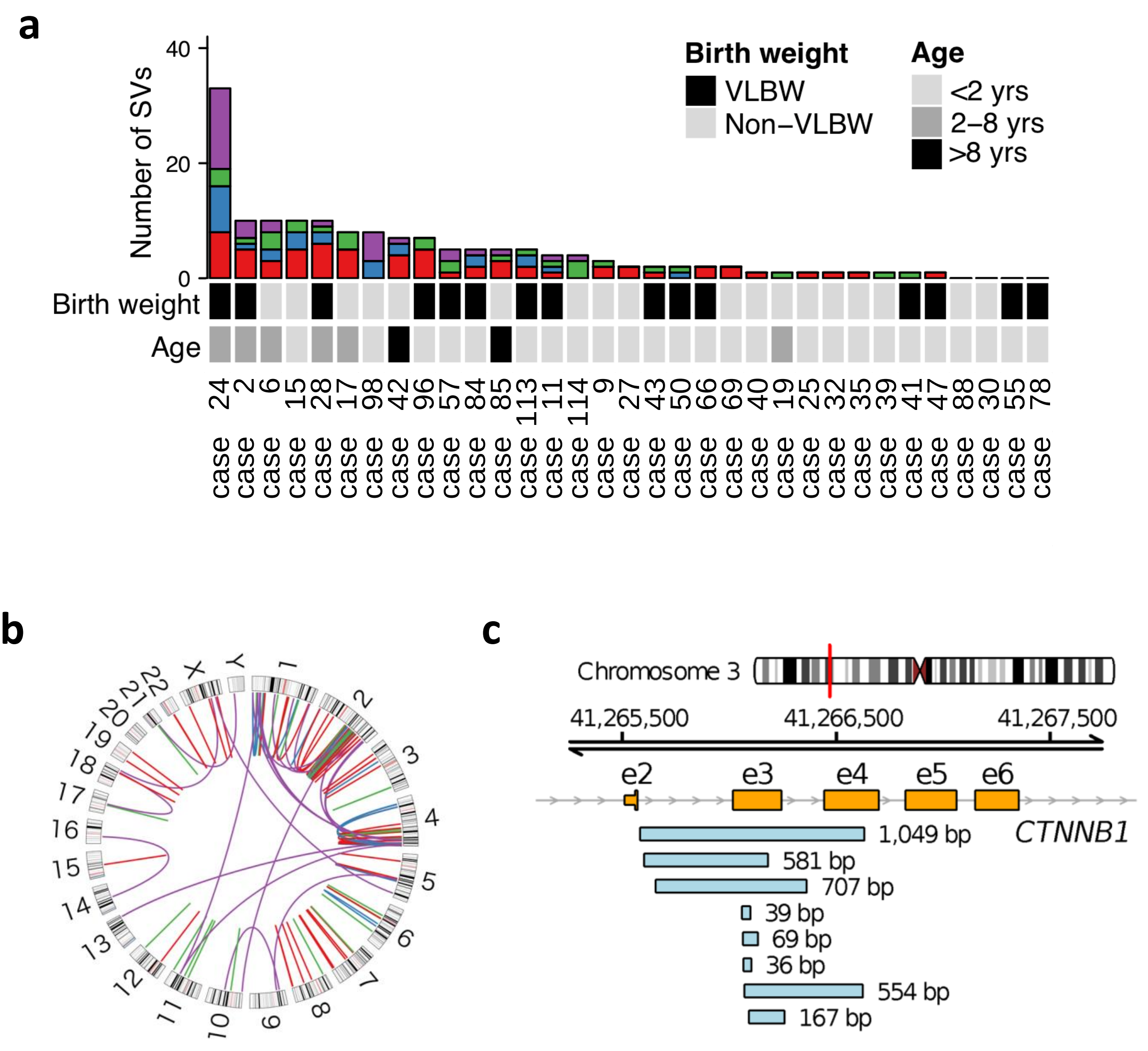

Supplementary Fig. 4: Somatic structural variants (SVs) of childhood hepatoblastoma (HB).

a, The number of somatic structural variants (SVs) identified by WGS of 33 HB. **b**, Circos plot for somatic SVs of 33 HB genomes. Chromosomes displaying cytobands are arranged end-to-end in the outer ring. Color code of inter-chromosomal rearrangements is displayed and is the same as shown in Supplementary Fig 4a. **c**, SVs in 3p22.1. Light blue bars represent deletions.

Supplementary Fig. 5

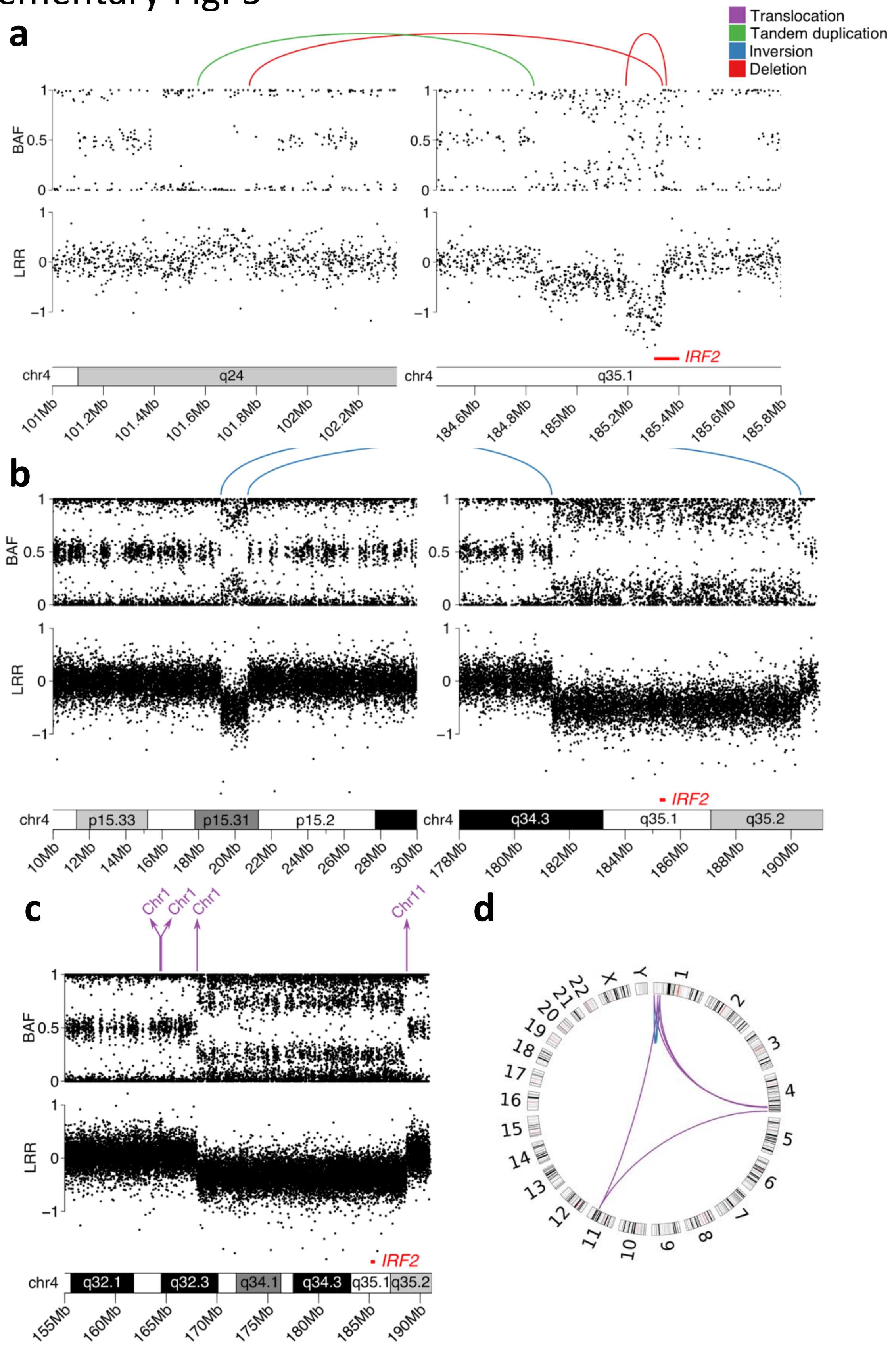

Supplementary Fig. 5: Somatic copy number alterations and structural variants in 4q35.1.
Log R ratio (LRR) and B allele frequency (BAF) were measured using SNP arrays. Structural variants were detected using whole-genome sequencing (WGS) and are shown as arcs and arrows. **a**, case 17. **b**, case 42. **c**, case 98. **d**, Circos plot for somatic structural variants (SVs) in case 98.

Supplementary Fig. 6

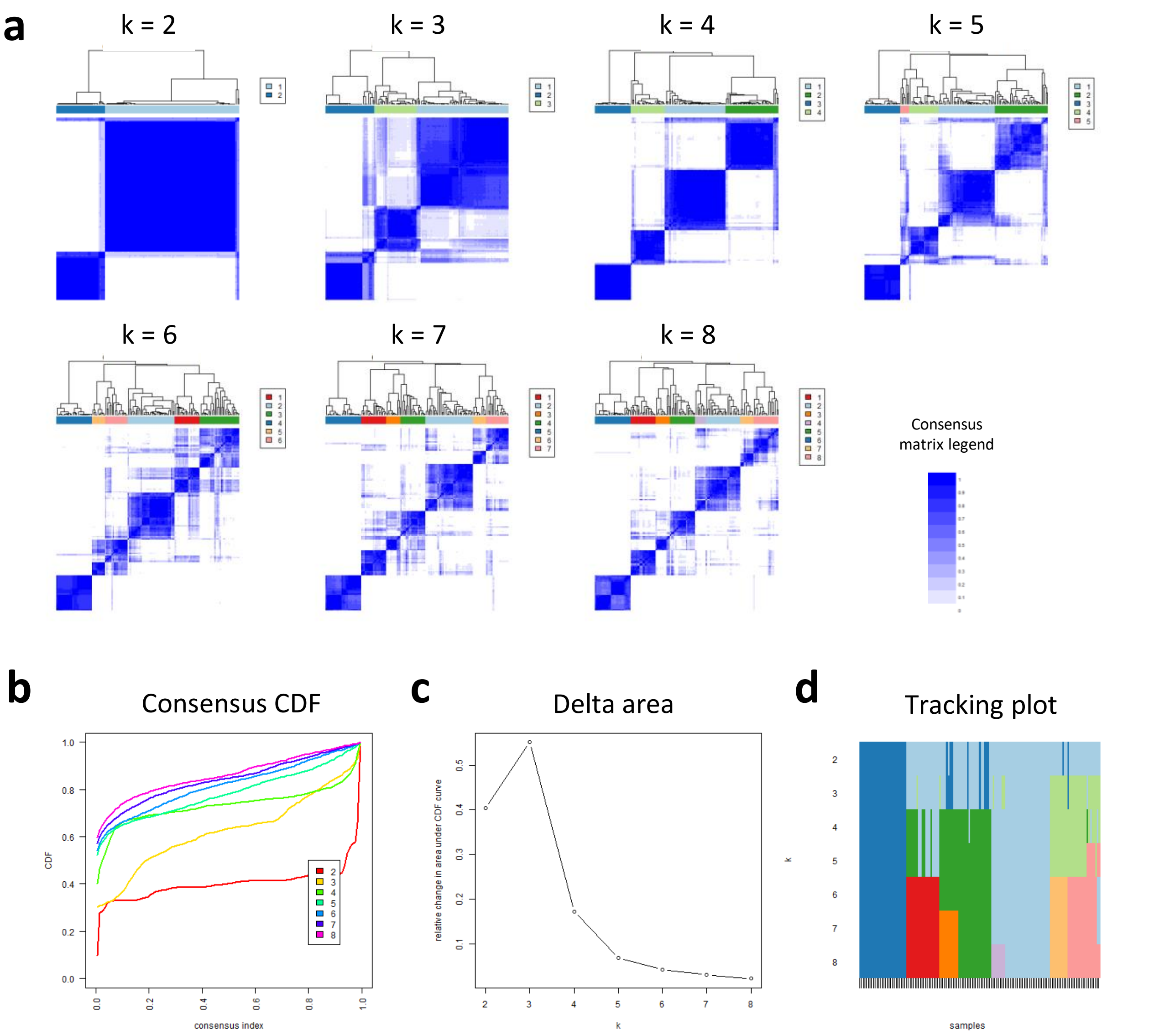

Supplementary Fig. 6: The robustness of the gene expression subtypes determined through Consensus Clustering.

a, The consensus matrix of 1000 times of repetition sampling for each k (k = 2~8) The consensus matrices have items as both rows and columns, which are RNA-sequencing in this example, and where consensus values range from 0 (never clustered together) to 1 (always clustered together) marked by white to dark blue. The consensus matrices are ordered by consensus clustering which is depicted as a dendrogram atop the heatmap. **b**, Consensus cumulative distribution curve (CDF) plots display consensus distributions for each k to find the k at which the distribution reaches an approximate maximum. **c**, Delta area shows the relative change in the area under the CDF curve to determine the relative increase in consensus and to determine the k value at which there is no appreciable increase. **d**, tracking plot shows the consensus cluster of items (in columns) at each k (in rows) to track an item's cluster assignments across different k values, to identify promiscuous items that are suggestive of weak class membership, and to visualize the distribution of cluster sizes across k.

Supplementary Fig. 7

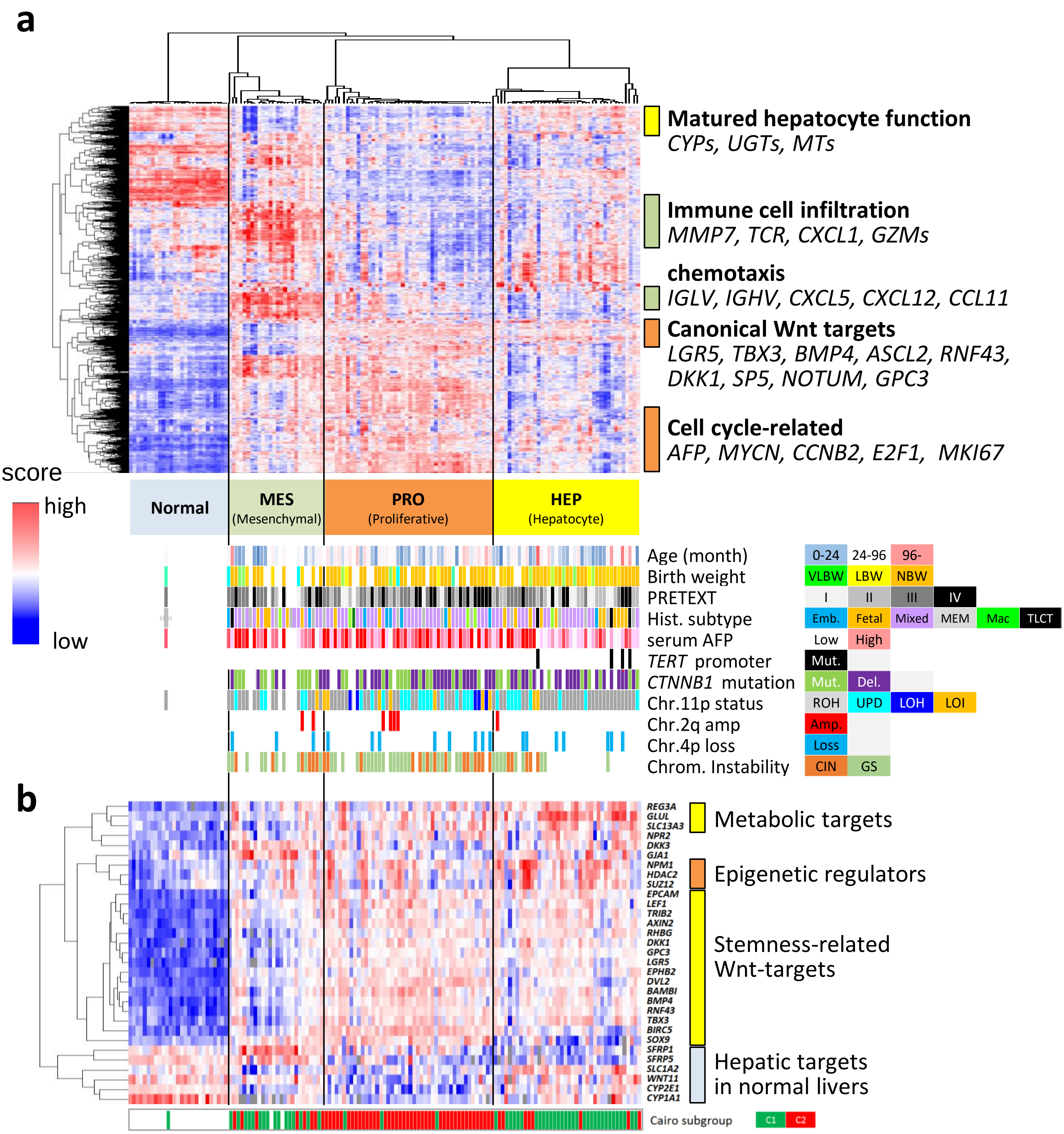

Supplementary Fig. 7

c

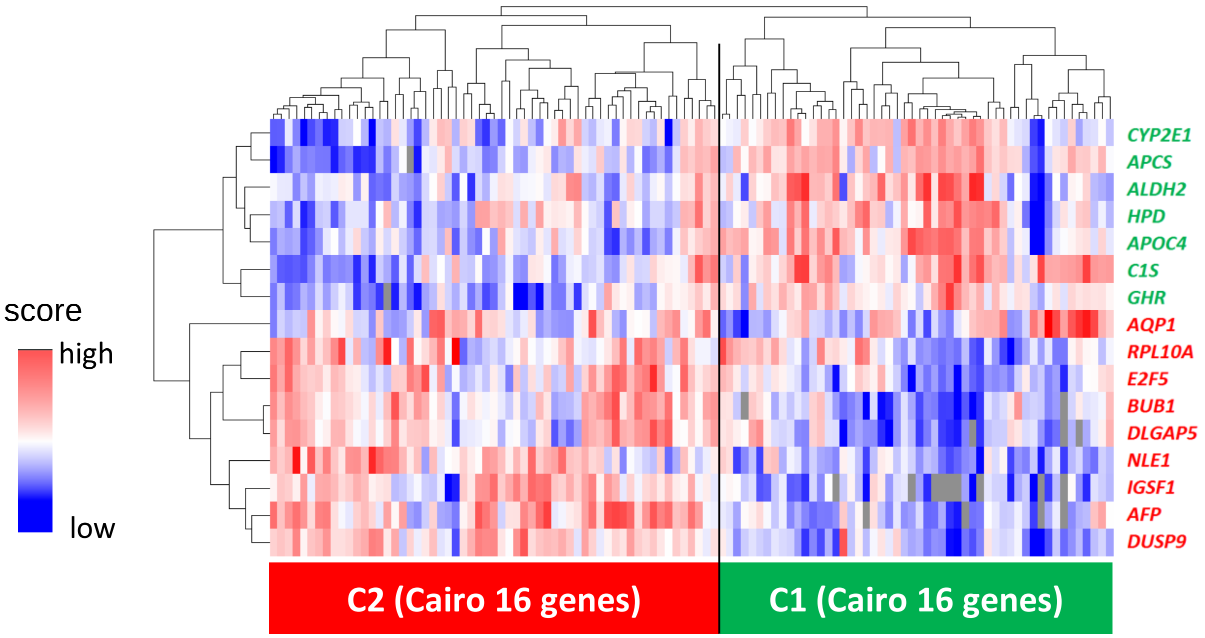

Supplementary Fig. 7: Gene expression subtyping of hepatoblastoma. **a**, The heat map in the top shows gene expression levels of each tumor (horizontal axis) plotted for the 5,000 most variably expressed genes (vertical axis). 135 samples are clustered into the four subgroups; "proliferative (PRO)" (n = 46), "hepatocyte (HEP)" (n = 40), "mesenchymal (MES)" (n = 26) and normal tissue (n=27) by consensus clustering (k = 4). The representative genes upregulated/downregulated in the specific subtypes are shown on the right. The middle panel shows the clinical and pathological parameters of HB patients. **b**, The heat map at the bottom represents the expression level of the 31 "hepatoblastoma genes"[2]. **c**, The clustering analysis of HB data using the "16-gene set" of hepatoblastoma genes[2]. Each marker gene is shown on the right side of the heatmap. The red and green bars represent the C1 and C2 subtypes, respectively.

Supplementary Fig. 8

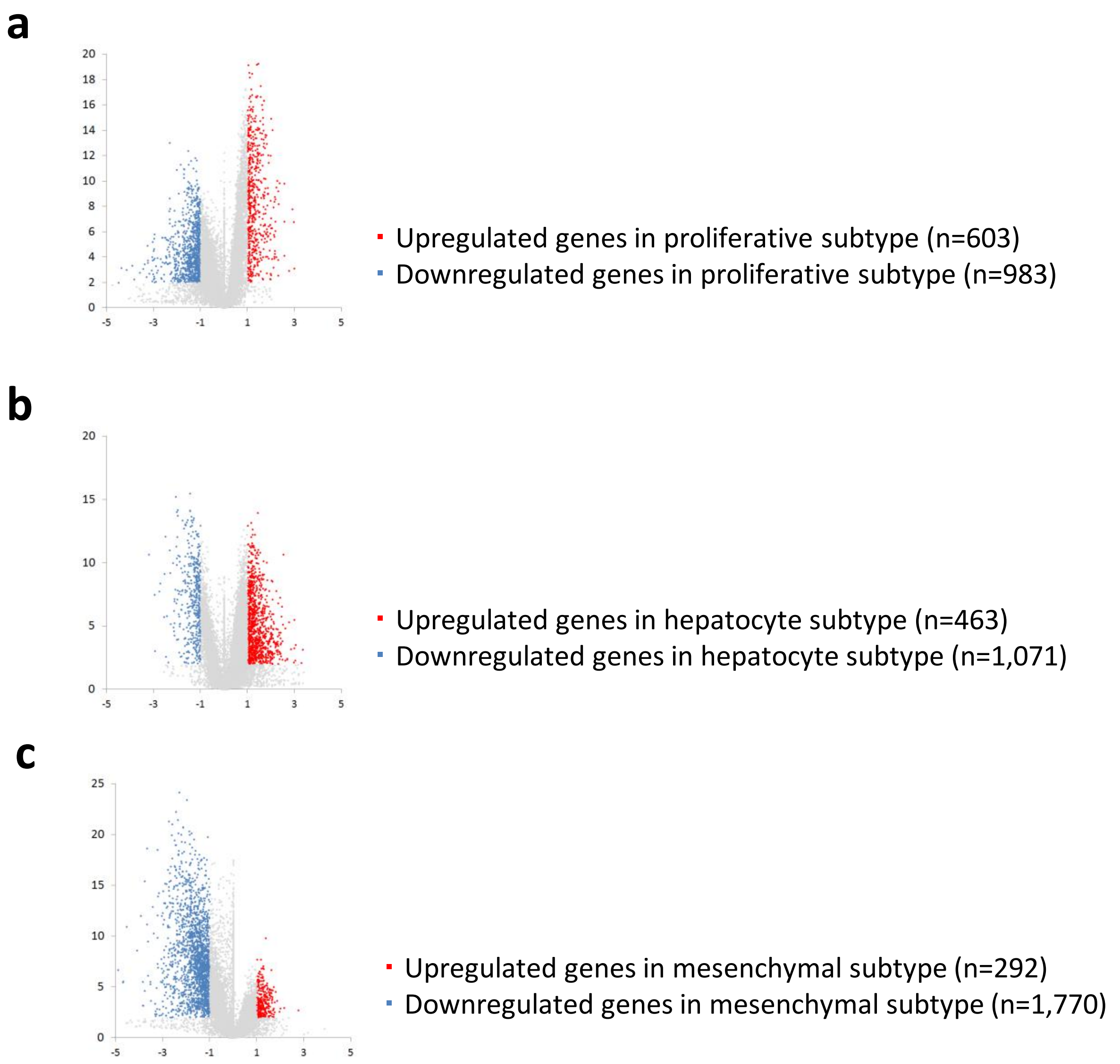

Supplementary Fig. 8: Differentially expressed genes among the three expression subtypes.
Volcano Plot for the visualization of the differentially expressed genes among the three expression subtypes (**a**, proliferative; **b**, hepatocyte; **c**, mesenchymal). The horizontal lines represent the fold-change values (log_2FC). The right vertical lines represent -log10 *p*-values calculated using two-sided t-test. The upregulated genes ($log_2FC > 1$, p-value < 0.01) and downregulated genes ($log_2FC < -1$, p-value < 0.01) are marked in red and blue, respectively. The 25 upregulated and downregulated genes in each gene expression subtype with the most fold-change value (log_2FC) are shown in the Supplementary dataset 3 .

Supplementary Fig. 9

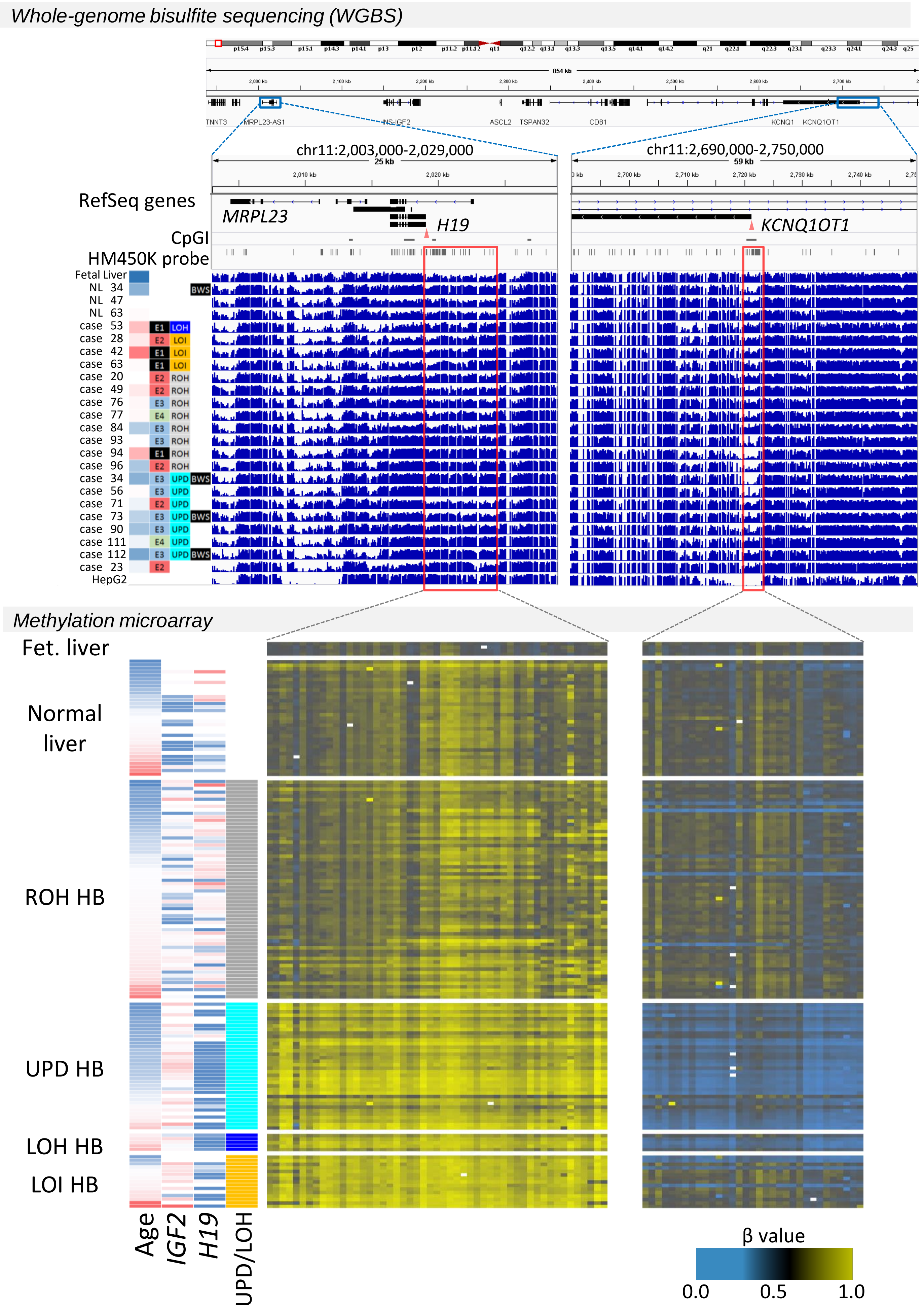

Supplementary Fig. 9: Methylation status of the imprinting control regions (ICRs) of *H19* and *KCNQ1OT1*.
The top panel represents cytosine methylation levels of two ICRs, 21 HBs, fetal liver, and HepG2 cell line by whole-genome bisulfite sequencing (WGBS). The heat map shows methylation statuses of childhood hepatoblastoma (HB) tumors with variable Chr.11p statuses (vertical axis) plotted by physical position around the ICRs for *H19* and *KCNQ1OT1* (horizontal axis).

Supplementary Fig. 10

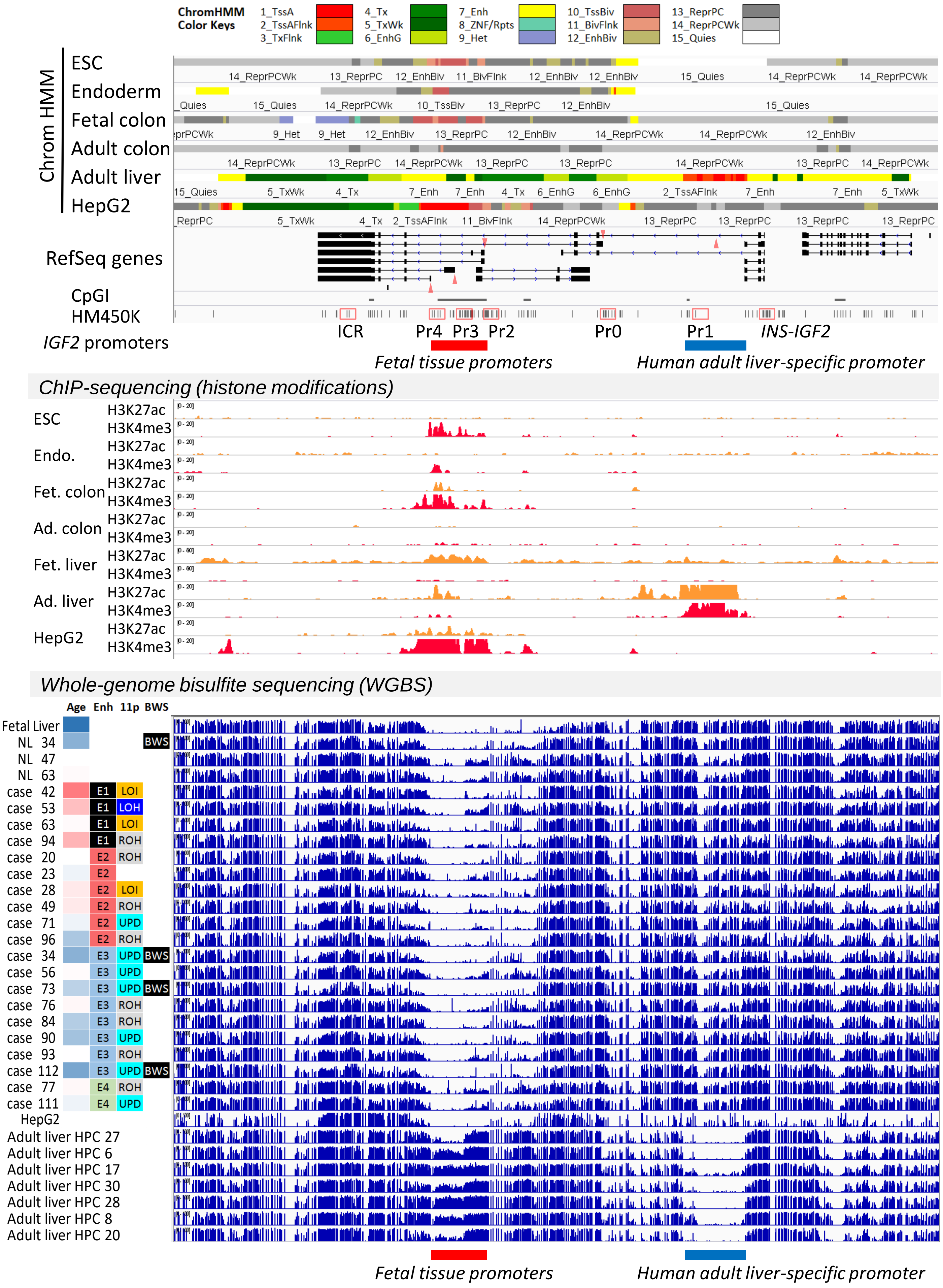

Supplementary Fig. 10: Epigenomic statuses of *IGF2* promoter regions.

The top panel represents chromatin statuses of human embryonic stem cells (H9, E003), their in vitro derivative cells toward endoderm lineage (E011), fetal colon (E084), adult colon (E106), and adult liver tissues (E066) as defined in the Roadmap Epigenomics project (https://egg2.wustl.edu/roadmap/web_portal/index.html) around the *IGF2* gene (chr11:2,140,000-2,195,000). 1_TssA (Red), Active TSS; 2_TssAFlnk (Orange Red), Flanking Active TSS; 3_TxFlnk (LimeGreen), Transcr. at gene 5' and 3'; 4_Tx (Green), Strong transcription; 5_TxWk (DarkGreen), Weak transcription; 6_EnhG (GreenYellow), Genic enhancers; 7_Enh (Yellow), Enhancers; 8_ZNF/Rpts (Medium Aquamarine), ZNF genes & repeats; 9_Het (Pale Turquoise), Heterochromatin; 10_TssBiv (IndianRed), Bivalent/Poised TSS; 11_BivFlnk (DarlSalmon), Flanking Bivalent TSS/Enh; 12_EnhBiv (DarkKhaki), Bivalent Enhancer; 13_ReprPC (Silver), Repressed PolyComb; 14_ReprPCWk (Gainsboro), Weak Repressed PolyComb; 15_Quies (White), Quiescent/Low. Red triangles represent the transcription start sites of five *IGF2* promoters. The middle panel shows histone modification statuses (H3K27ac, H3K4me3) in each samples of the Roadmap Epigenomics project and the published data of fetal liver[3] (H3K27ac, GSM1598036; H3K4me3, GSM1598044). The bottom panel represents cytosine methylation levels examined by whole-genome bisulfite sequencing (WGBS) for the fetal liver, 3 normal liver, 20 HB in this study, and for HepG2 and the 7 adult livers in the IHEC data portal (https://epigenomesportal.ca/ihec/download.html#). Clinical and molecular parameters (Age, age at diagnosis; Enh, Enhancer methylation subtype; 11p, copy number status of chromosome 11p; BWS, Beckwith-Wiedemann syndrome) are shown at the left of the bar graph.

Supplementary Fig. 11

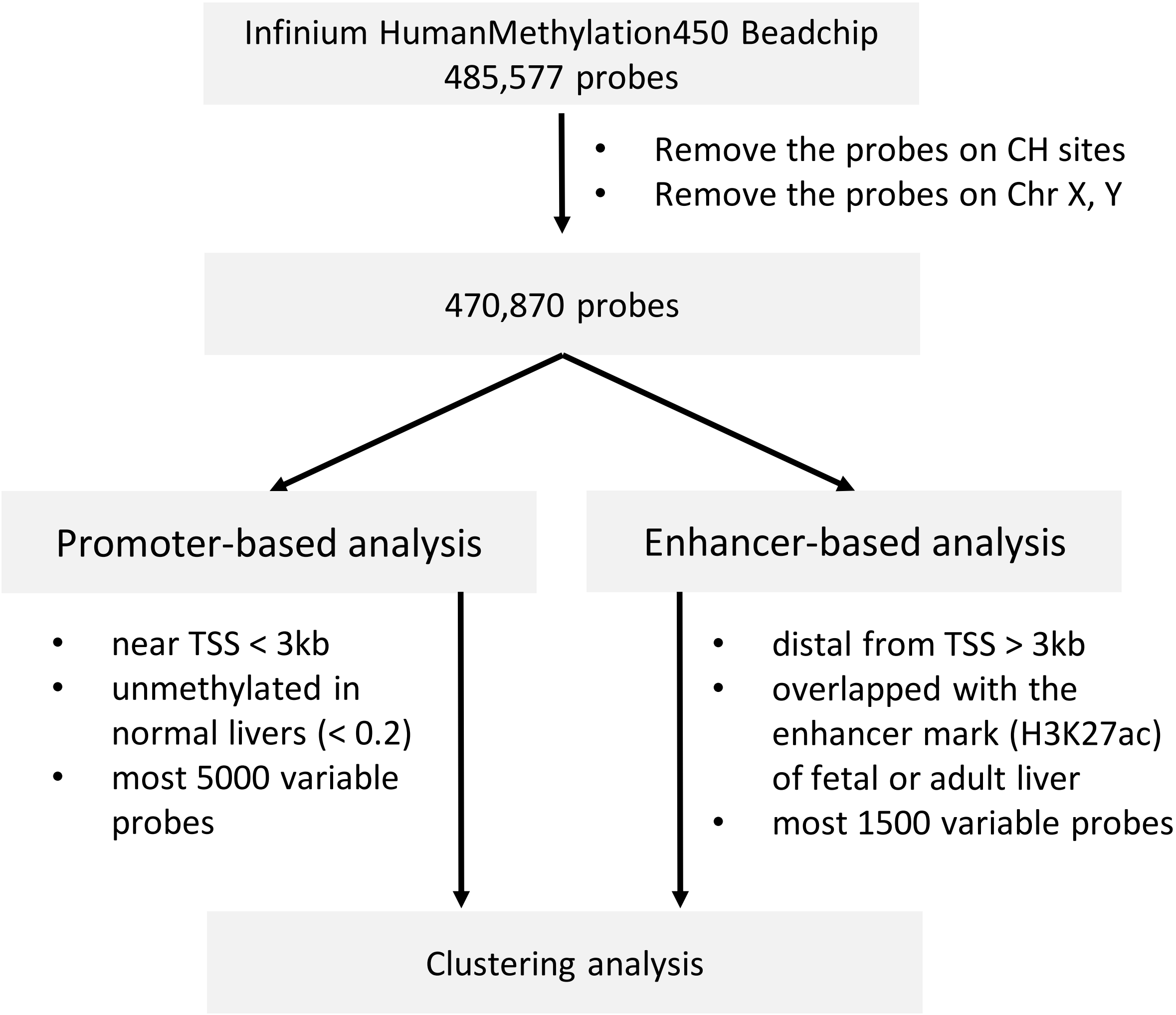

Supplementary Fig. 11: Probe selection for global methylation analysis.

Supplementary Fig. 12

a

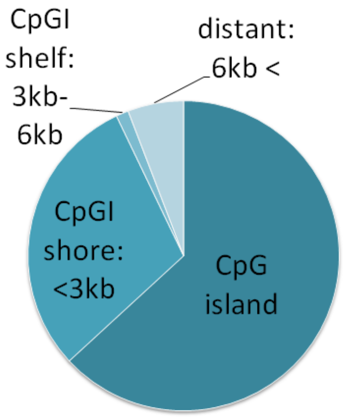

b

	ESC	ENDO	LIV.ADLT	HepG2
1_TssA	1526	1548	880	408
2_TssAFlnk	260	115	1170	348
3_TxFlnk	17	37	5	25
4_Tx	4	14	1	64
5_TxWk	30	151	180	632
6_EnhG	1	0	5	6
7_Enh	65	78	596	158
8_ZNF/Rpts	103	75	258	9
9_Het	105	131	70	224
10_TssBiv	1378	1064	66	310
11_BivFlnk	822	217	132	198
12_EnhBiv	364	252	523	73
13_ReprPC	177	826	526	761
14_ReprPCWk	48	215	425	1061
15_Quies	100	277	163	723

ESC — human embryonic stem cells (hESC), H1
ENDO — endoderm differentiation from hESC
LIV.ADLT — Adult Liver
HepG2 — HepG2, hepatoblastoma cell line

c

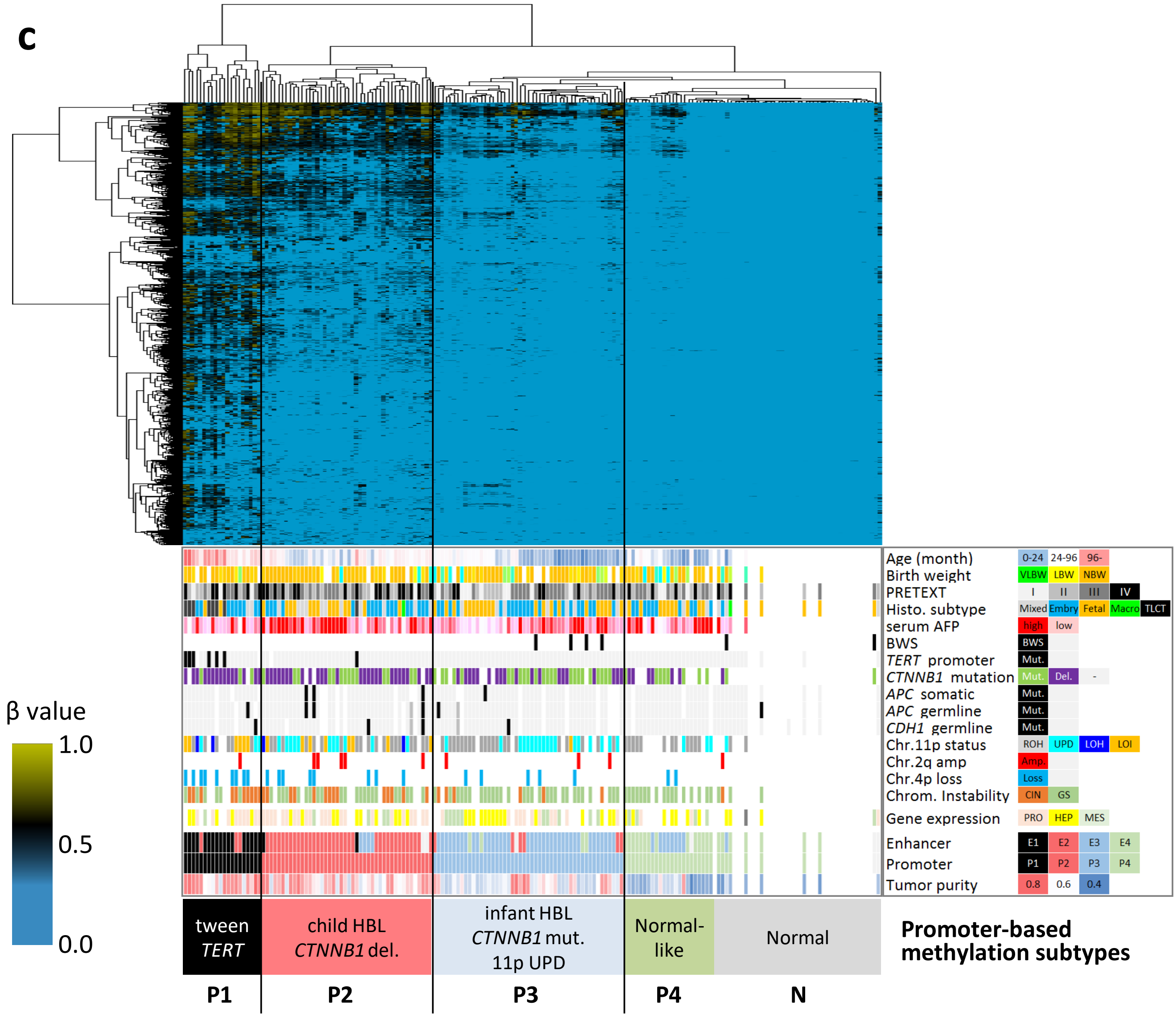

Supplementary Fig. 12: Promoter methylation subtypes of hepatoblastoma.
a, distance distribution of 5000 selected probes from the CpG island of the human genome. **b**, chromatin statuses of ESC (human embryonic stem cell, H1), ENDO (endoderm differentiation from hESC), and LIV.ADLT (adult liver) and HepG2 in the 5000 selected probes. **c**, Unsupervised clustering of 146 childhood hepatoblastoma (HB) tumors and 11 non-cancerous livers using the 5,000 most variably methylated CpGs located at the promoter regions. The heat map shows the methylation level of each tumor (horizontal axis) plotted by the probe sets (vertical axis) in the top panel. The bottom panel shows clinical and pathological parameters, and the molecularly-defined subgroups of HB patients.

Supplementary Fig. 13

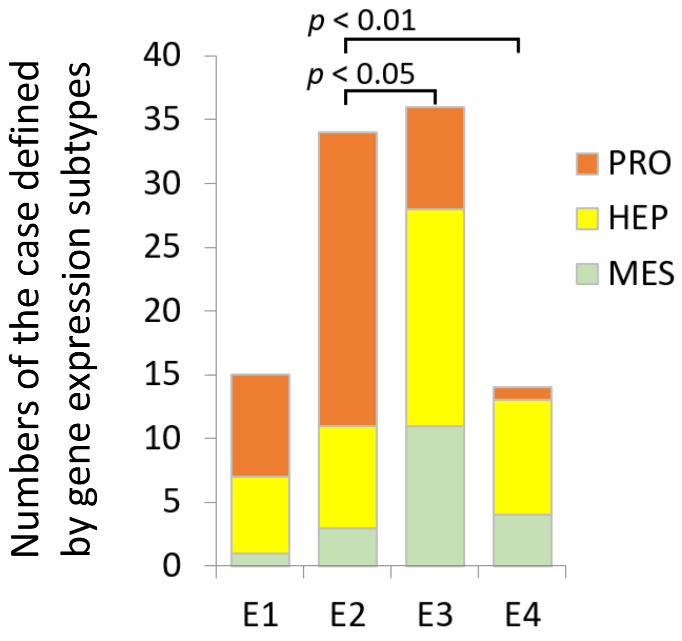

Supplementary Fig. 13: Comparison analysis of the gene expression subtypes and enhancer methylation subtypes.
Bar graph represents the number of cases defined by gene expression subtypes. Pairwise comparisons (a comparison of the proportions of these 2 categorical variables) were performed using Fisher's exact test.

Supplementary Fig. 14

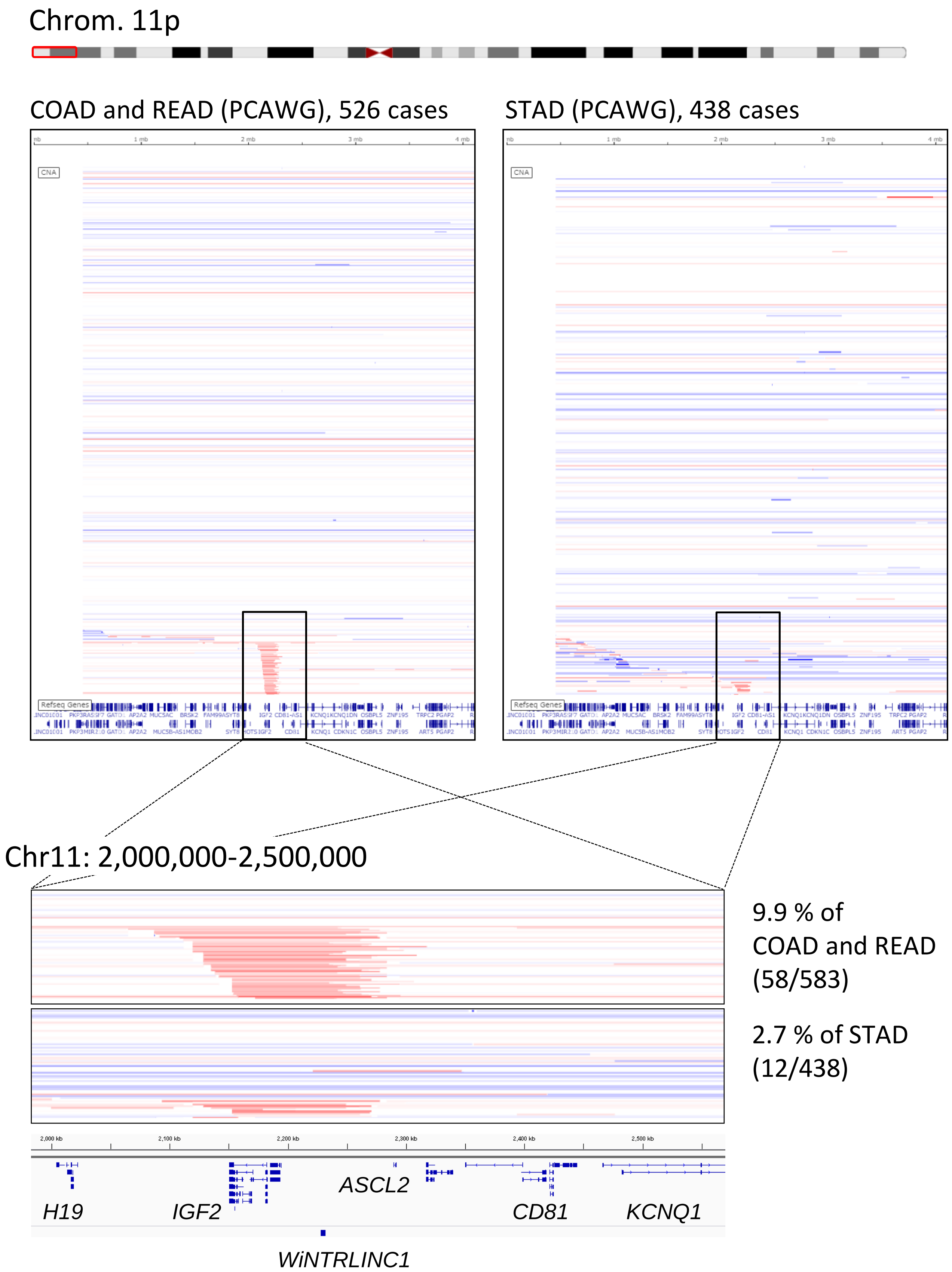

Supplementary Fig. 14: Local amplification in *IGF2/ASCL2* locus. Recurrent focal amplification in *IGF2/ASCL2* region of colorectal carcinoma (COAD, READ) and stomach adenocarcinoma (STAD) published in TCGA public database (cBioPortal, https://www.cbioportal.org/).

Supplementary Fig. 15

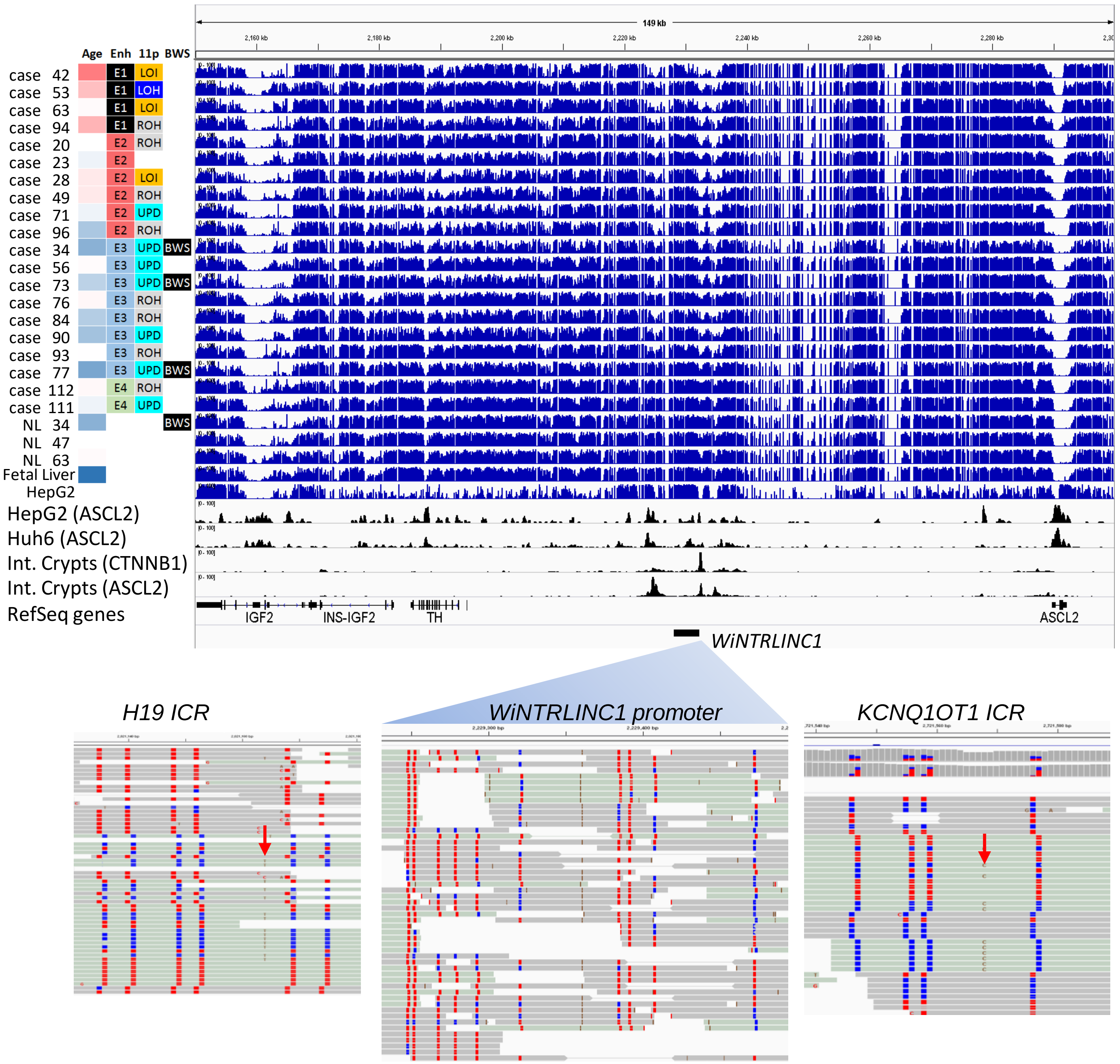

Supplementary Fig. 15: Allele-specific and -unspecific methylation at IGF2/ASCL2 locus.

Landscape of methylation statues in IGF2/ASCL2 regions analyzed by whole-genome bisulfite sequencing (blue bar graphs, 21 HB, 3 normal livers, and the liver cancer cell line, HepG2). The Latter bars show the sequencing reads representing the methylation status of each CpG site (red, methylated; blue, unmethylated; A/C/G/T, germline SNPs). Red arrows represent typical examples of the germline SNPs. The panel shows the original top strand (OT), the original bottom strand (OB), and strands that are complementary to OT and OB (CTOT and CTOB) in WGBS data. OT and CTOT reads are displayed in the reference-forward direction (gray) while OB and CTOB reads are displayed in the reverse direction (sage) and are differentially colored as indicated.

Supplementary Fig. 16

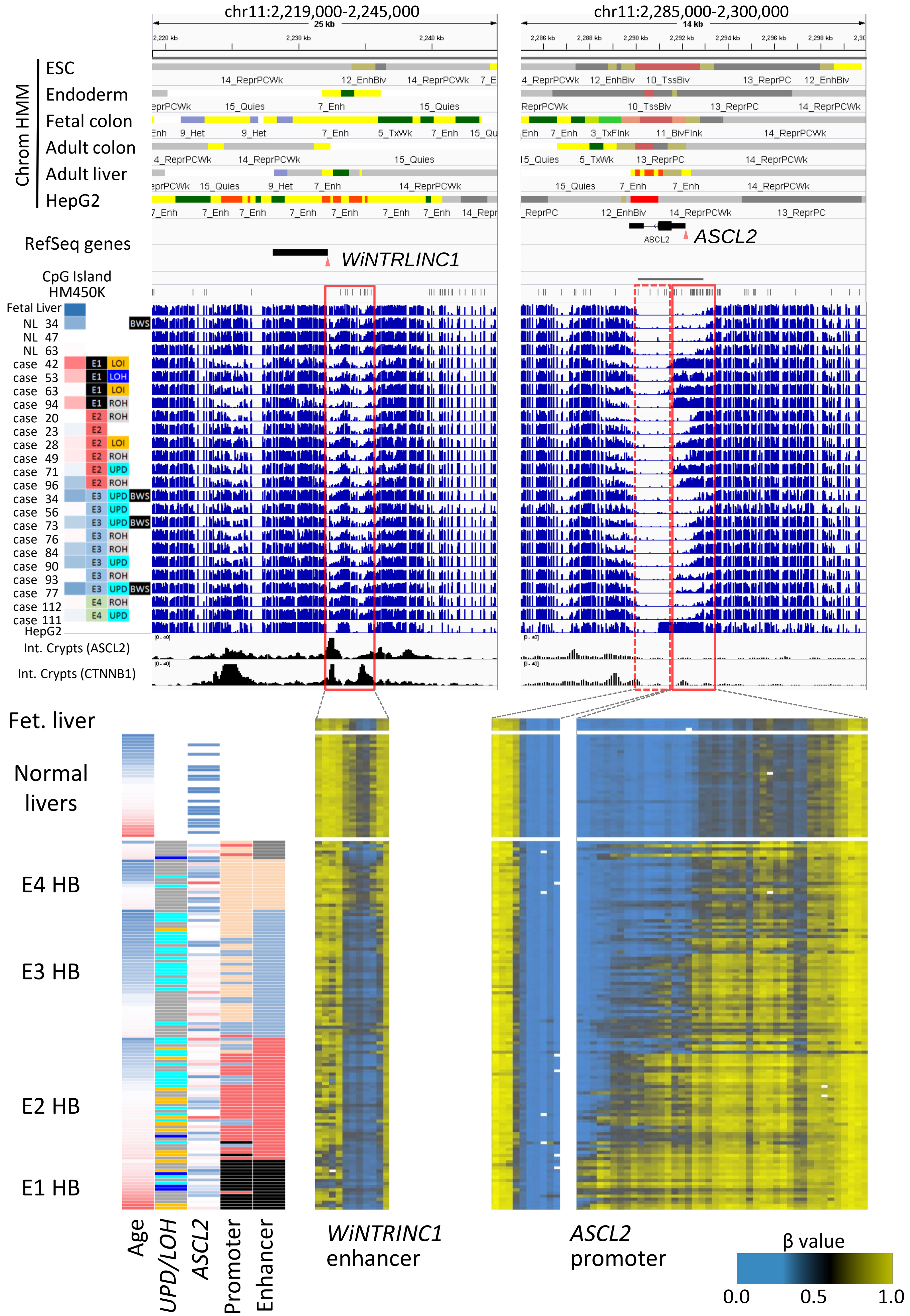

Supplementary Fig. 16: Methylation status of *WiNTRLINC1* and *ASCL2* in hepatoblastoma.

The top panel represents chromatin statuses of human embryonic stem cells (H9, E003), their in vitro derivative cells toward endoderm lineage (E011), fetal colon (E084), adult colon (E106), and adult liver tissues (E066) defined in the Roadmap Epigenomics project (https://egg2.wustl.edu/roadmap/web_portal/index.html) around the *WiNTRLINC1* gene (chr11:2,219,000-2,245,000) and *ASCL2* gene (chr11:2,285,000-2,300,000). 1_TssA (Red), Active TSS; 2_TssAFlnk (Orange Red), Flanking Active TSS; 3_TxFlnk (LimeGreen), Transcr. at gene 5' and 3‘; 4_Tx (Green), Strong transcription; 5_TxWk (DarkGreen), Weak transcription; 6_EnhG (GreenYellow), Genic enhancers; 7_Enh (Yellow), Enhancers; 8_ZNF/Rpts (Medium Aquamarine), ZNF genes & repeats; 9_Het (Pale Turquoise), Heterochromatin; 10_TssBiv (IndianRed), Bivalent/Poised TSS; 11_BivFlnk (DarlSalmon), Flanking Bivalent TSS/Enh; 12_EnhBiv (DarkKhaki), Bivalent Enhancer; 13_ReprPC (Silver), Repressed PolyComb; 14_ReprPCWk (Gainsboro), Weak Repressed PolyComb; 15_Quies (White), Quiescent/Low. Red triangles represent the transcription start sites of *WiNTRLINC1* and *ASCL2*. The middle panel represents cytosine methylation levels in the fetal liver, 3 normal livers, 20 HB, and the HepG2 cell line, examined by whole-genome bisulfite sequencing (WGBS) in this study. The heat map in the bottom panel represents cytosine methylation levels across 146 HB and 13 normal livers.

Supplementary Fig. 17

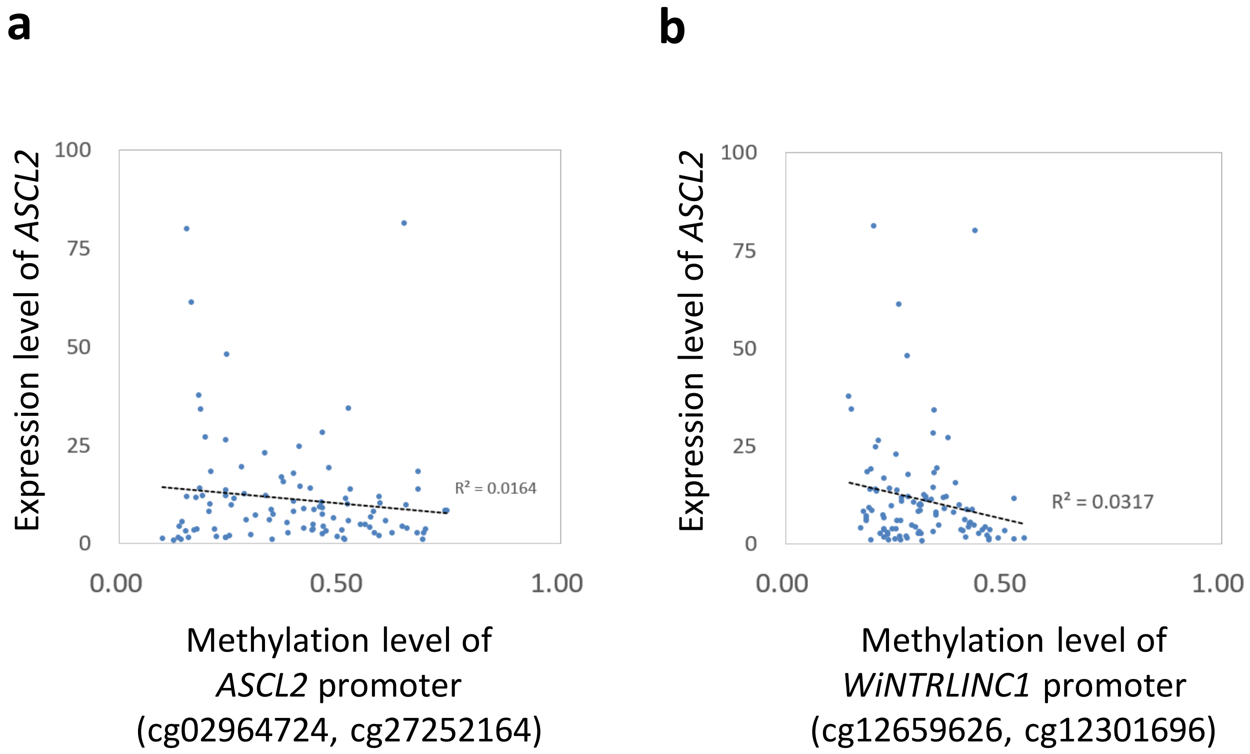

Supplementary Fig. 17: Relationship between gene expression and methylation in ASCL2.
Scatter plots represent the relationship between the gene expression level of *ASCL2* and the average methylation level of the promoter region (*ASCL2*: cg02964724, cg27252164; *WiNTRLINC1*: cg12659626, cg12301696). The dashed line represents a linear regression. Source data are provided as a Source Data file.

Supplementary Fig. 18

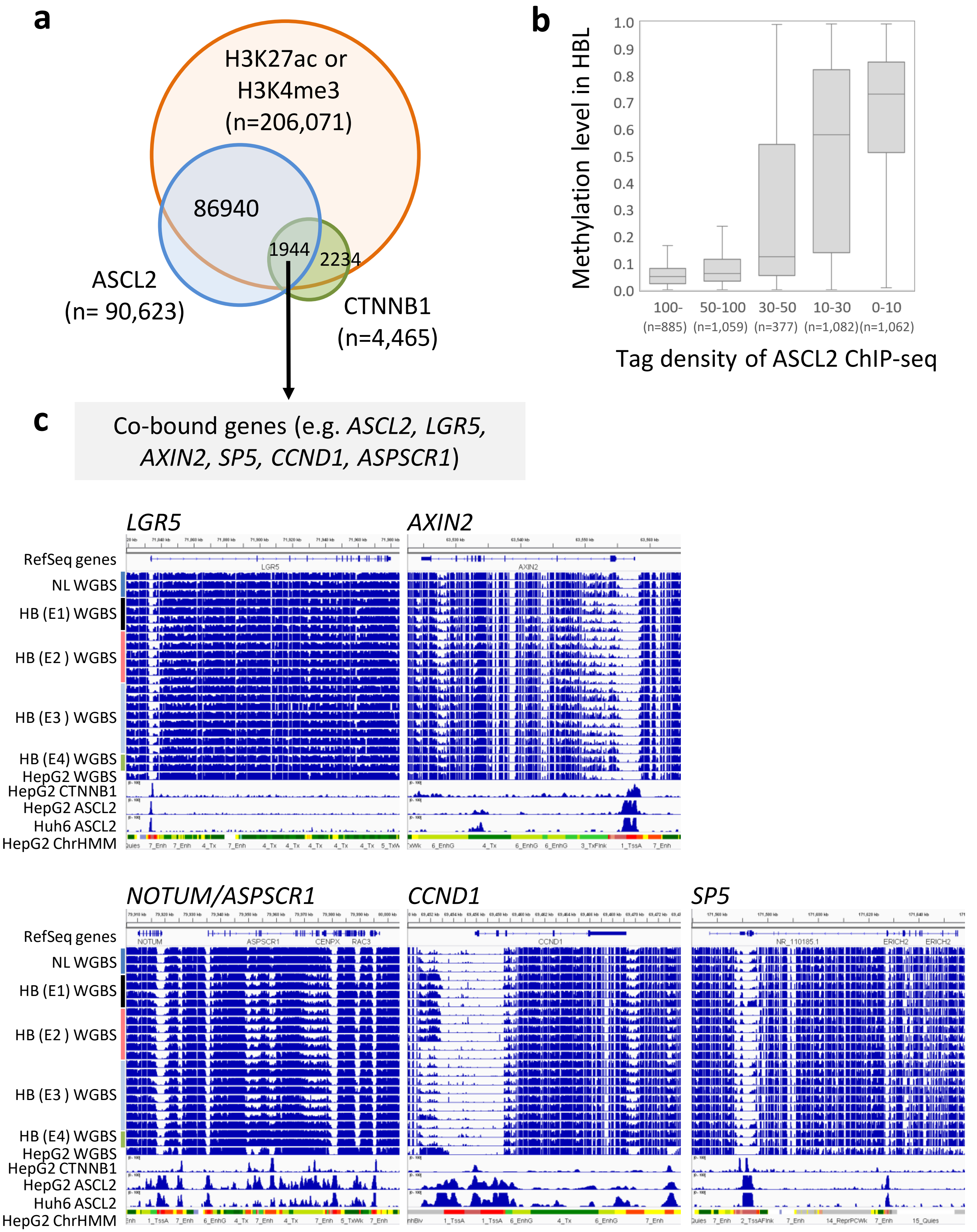

Supplementary Fig. 18: Genome-wide binding of ASCL2 and CTNNB1 in hepatoblastoma (HB).

a, Venn diagram showing the overlap of ASCL2-bound (blue), CTNNB1-bound (green), and active chromatin regions (red) in HepG2.

b, Global distribution of the average methylation level in 146 HB samples along with the strength of the endogenous ASCL2 binding (tag density of ChIP-sequencing against ASCL2). The center lines show the medians, the tops and bottoms of boxes show quartiles, and the whiskers show the extremes within the range of the medians ± 1.5 × the interquartile ranges. Source data are provided as a Source Data file.

c, Representative Wnt-targeted genes co-bound with ASCL2 and CTNNB1 (*LGR5*, *AXIN2*, *NOTUM,* and *ASPSCR1*, *CCND1*, *SP5*).

Supplementary Fig. 19

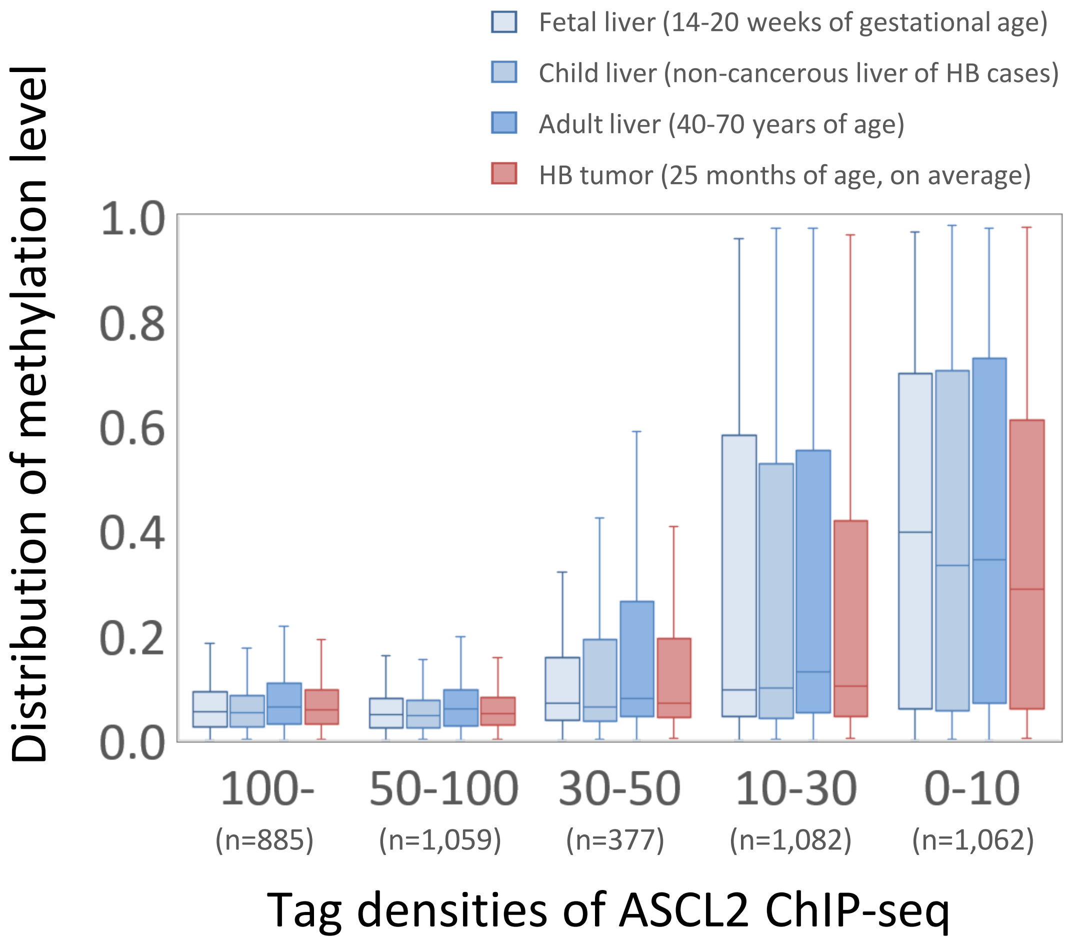

Supplementary Fig. 19: The differentially methylated regions between fetal and adult liver tissues
Global distribution of the average methylation level in non-cancerous liver samples along with the strength of the endogenous ASCL2 binding (tag density of ChIP-sequencing against ASCL2). The center lines show the medians, the tops and bottoms of boxes show quartiles, and the whiskers show the extremes within the range of the medians ± 1.5 × the interquartile ranges. Source data are provided as a Source Data file.

Supplementary Fig. 20

a

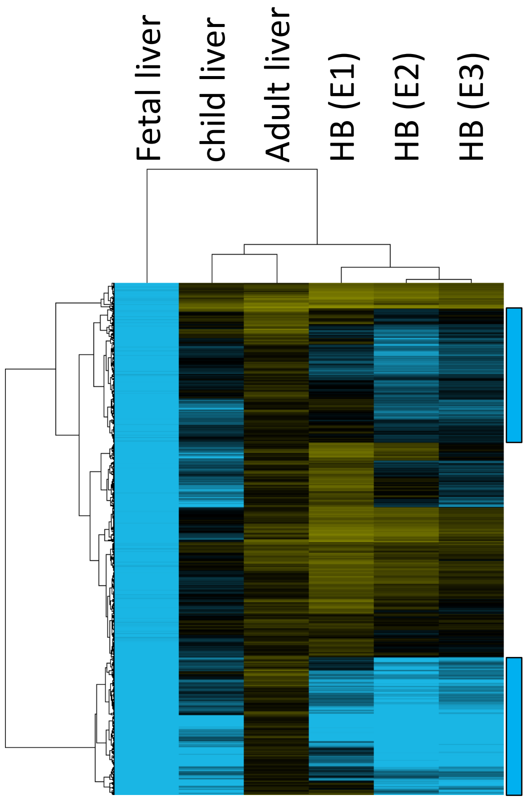

Hypermethylation in adult liver:
INS-IGF2, IGF2AS, ESR1, PPT2, CYBA, TIAM1, B3GALT4, DEF6, EFCAB4B, GRHL3, TRIOBP, APBB2, NR4A2, SRCIN1, ZNF385A, DAB2IP, GFRA3, NR2F2, SLC16A5, TET1, C10orf41, CNTN2, DCBLD1, DTX1, FOXD2, KCNQ4, SP5, ST8SIA3, AGRN, AMTA1, DKFZP434H168, FGF2, GHRLOS, HAPLN4, MACROD1, MAP3K13, MEIS2, MGC12982, MYADM, RARA, SLC16A3, SNED1, SOX7, SSBP4, TRAF3IP2, TSPAN9, ZMIZ1, ZNF254

b

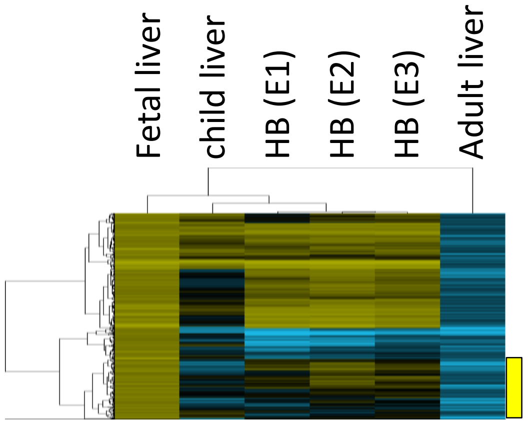

Hypomethylation in fetal liver:
COMT, PCCA, ZC3H3, BAIAP2, BANP, C21orf70, C4BPA, CUGBP1, DAXX, DUSP1, EMP2, MPST, MYH9, NADK, NEK3, PPP1R3C, STK24

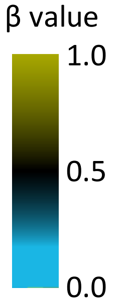

Supplementary Fig. 20: Comparative analysis of fetal-liver-specific hypermethylation and hypomethylation among non-cancerous liver samples.
Fetal-liver-specific hypermethylation (a) and hypomethylation (b) are selected by the difference in beta values (fetal liver – adult liver > 0.3, <-0.3, respectively). The heat map shows the average methylation level among the clinical tissue samples. Recurrently listed genes are shown in gray boxes in the right.

Supplementary Fig. 21

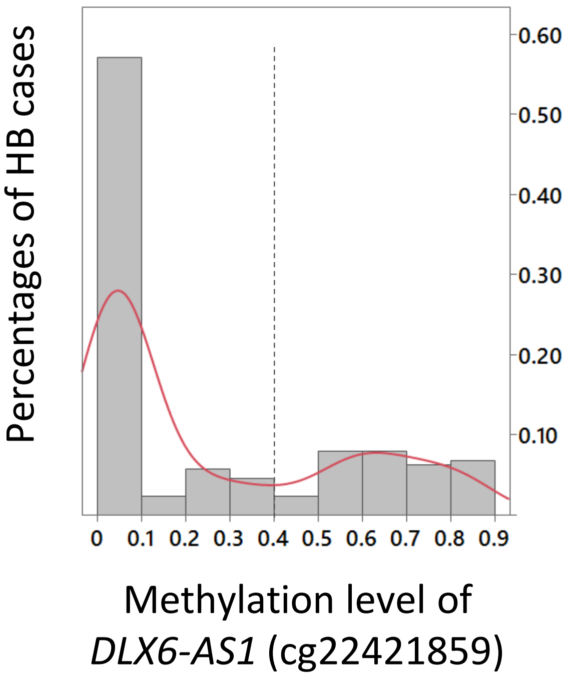

Supplementary Fig. 21: Distribution of *DLX6AS* methylation
Histogram (gray bar) of *DLX6-AS1* methylation (cg22421859) and probability density estimated via Kernel density estimation (red line). The black dashed line represents a cut-off to discriminate between methylated (M) and unmethylated (U). Source data are provided as a Source Data file.

Supplementary References

1. Lopez-Terrada, D. *et al*. Towards an international pediatric liver tumor consensus classification: proceedings of the Los Angeles COG liver tumors symposium. *Mod Pathol* **27**, 472–491 (2014).

2. Cairo, S. *et al*. Hepatic stem-like phenotype and interplay of Wnt/beta-catenin and Myc signaling in aggressive childhood liver cancer. *Cancer Cell* **14**, 471–484 (2008).

3. Yan L, *et al*. Epigenomic landscape of human fetal brain, heart, and liver. *J Biol Chem* **291**:4386-98 (2016)
