# Supplementary material for: Genetic and epigenetic basis of hepatoblastoma diversity
Source: Nat Commun. 2021 Sep 20;12:5423. doi: 10.1038/s41467-021-25430-9 (PMC8450290; doi:10.1038/s41467-021-25430-9)
Supplement: Supplementary file 3 — Description of Additional Supplementary Files [file 41467_2021_25430_MOESM3_ESM.pdf]

Description of Additional Supplementary Files

File Name: Supplementary Data 1
Description: Clinical information of 163 childhood liver cancer cohorts.

File Name: Supplementary Data 2
Description: GO terms related to expression subtypes

File Name: Supplementary Data 3
Description: Differentially expressed genes among the three expression subtypes (**a**, proliferative; **b**, hepatocyte; **c**, mesenchymal) using Volcano Plot analysis. The upper and lower tables represent 25 upregulated and downregulated genes in each gene expression subtype with the most fold-change value (log_2FC)
