# Supplementary material for: Genetic and epigenetic basis of hepatoblastoma diversity
Source: Nat Commun. 2021 Sep 20;12:5423. doi: 10.1038/s41467-021-25430-9 (PMC8450290; doi:10.1038/s41467-021-25430-9)
Supplement: Supplementary file 7 — Reporting Summary [file 41467_2021_25430_MOESM7_ESM.pdf]

nature research

Corresponding author(s): Eiso Hiyama
Hiroyuki Aburatani
Hidewaki Nakagawa

Last updated by author(s): Aug 6, 2021

Reporting Summary

Nature Research wishes to improve the reproducibility of the work that we publish. This form provides structure for consistency and transparency in reporting. For further information on Nature Research policies, see our Editorial Policies and the Editorial Policy Checklist.

Statistics

For all statistical analyses, confirm that the following items are present in the figure legend, table legend, main text, or Methods section.

n/a	Confirmed	
☐	☒	The exact sample size (*n*) for each experimental group/condition, given as a discrete number and unit of measurement
☐	☒	A statement on whether measurements were taken from distinct samples or whether the same sample was measured repeatedly
☐	☒	The statistical test(s) used AND whether they are one- or two-sided<br>*Only common tests should be described solely by name; describe more complex techniques in the Methods section.*
☐	☒	A description of all covariates tested
☐	☒	A description of any assumptions or corrections, such as tests of normality and adjustment for multiple comparisons
☐	☒	A full description of the statistical parameters including central tendency (e.g. means) or other basic estimates (e.g. regression coefficient) AND variation (e.g. standard deviation) or associated estimates of uncertainty (e.g. confidence intervals)
☐	☒	For null hypothesis testing, the test statistic (e.g. *F*, *t*, *r*) with confidence intervals, effect sizes, degrees of freedom and *P* value noted<br>*Give P values as exact values whenever suitable.*
☒	☐	For Bayesian analysis, information on the choice of priors and Markov chain Monte Carlo settings
☐	☒	For hierarchical and complex designs, identification of the appropriate level for tests and full reporting of outcomes
☒	☐	Estimates of effect sizes (e.g. Cohen's *d*, Pearson's *r*), indicating how they were calculated

Our web collection on statistics for biologists contains articles on many of the points above.

Software and code

Policy information about availability of computer code

Data collection	No software was used for data collection.
Data analysis	The following publicly available software was used for analysis:<br>BWA-0.7.8 (for WGS), GenomonSV (v0.4.2rc), SignatureAnalyzer (v1.1), deconstructSigs (v1.9.0), BWA (0.5.9-r16, 0.7.12-r1039 for WXS), karkinos (in-house pipeline, v4.1.11, https://github.com/genome-rcast/karkinos), Genome Analysis Toolkit (v3.6), Affymetrix Power Tools (v1.21.0), GEMCA (in-house pipeline, Komura et al. Genome Res. 2006), GISTIC 2.0 (v2.0.23), ASCAT (v.2.5.2), Genome Analysis Toolkit (v3.6), BWA (0.7.12-r1039 for RNA-seq), DAVID bioinformatics software (v6.7), GenomeStudio (v2011.1), Cluster 3.0 (v1.52), TRAP, Bowtie (v1.2.1.1 for ChIP-seq), deepTools-bamCoverage (v2.5.4), Bismark (v0.14.3), Bowtie2 (v2.2.5 for WGBS), Integrative Genomics Viewer (v2.3.98), R (v3.5.3) , RStudio (1.2.1335), JMP Pro 11.

For manuscripts utilizing custom algorithms or software that are central to the research but not yet described in published literature, software must be made available to editors and reviewers. We strongly encourage code deposition in a community repository (e.g. GitHub). See the Nature Research guidelines for submitting code & software for further information.

Data

Policy information about availability of data

All manuscripts must include a data availability statement. This statement should provide the following information, where applicable:

- Accession codes, unique identifiers, or web links for publicly available datasets
- A list of figures that have associated raw data
- A description of any restrictions on data availability

The expression level of ASCL2 in adult cancers in TCGA Pan-Cancer transcriptome data (https://portal.gdc.cancer.gov) is available at the GDC portal site (https://portal.gdc.cancer.gov/query?filters=%7B%22op%22%3A%22and%22%2C%22content%22%3A%5B%7B%22op%22%3A%22and%22%2C%22content%22%3A%5B%7B

%22op%22%3A%22in%22%2C%22content%22%3A%7B%22field%22%3A%22cases.project.program.name%22%2C%22value%22%3A%5B%22TCGA%22%5D%7D%7D%2C%7B%22op%22%3A%22and%22%2C%22content%22%3A%5B%7B%22op%22%3A%22in%22%2C%22content%22%3A%7B%22field%22%3A%22cases.samples.sample_type_id%22%2C%22value%22%3A%5B%2201%22%2C%2202%22%2C%2203%22%2C%2204%22%2C%2205%22%2C%2206%22%2C%2207%22%2C%2208%22%2C%2209%22%5D%7D%7D%2C%7B%22op%22%3A%22in%22%2C%22content%22%3A%7B%22field%22%3A%22files.analysis.workflow_type%22%2C%22value%22%3A%5B%22HTSeq%20-%20Counts%22%5D%7D%7D%5D%7%5D%7D%5D%7D&query=cases.project.program.name%20in%20%5B%22TCGA%22%5D%20and%20%20cases.samples.sample_type_id%20in%20%5B%2201%22%2C%20%2202%22%2C%20%2203%22%2C%2204%22%2C%2205%22%2C%2206%22%2C%2207%22%2C%2208%22%2C%2209%22%5D%20and%20files.analysis.workflow_type%20in%20%5B%22HTSeq%20-%20Counts%22%5D). The H3K27ac chromatin immunoprecipitation (ChIP)-seq data of adult (GSE96504, https://www.ncbi.nlm.nih.gov/geo/query/acc.cgi?acc=GSE96504) and fetal livers (GSM1598036, https://www.ncbi.nlm.nih.gov/geo/query/acc.cgi?acc=GSM1598036) in ENCODE Roadmap Project. The raw and processed sequence data of WGS are available under restricted access, access can be obtained by contacting the National Bioscience Database Center (NBDC) Human Database (hum0161: https://humandbs.biosciencedbc.jp/en/hum0161-v1). The raw and processed sequence data of WXS (Exome), RNA-seq, WGBS and microarray data (SNP array and methylation array) are available under restricted access, access can be obtained by contacting the NBDC Human Database (hum0233: https://humandbs.biosciencedbc.jp/en/hum0233-v1). The ChIP-sequencing data generated in this study are publicly available in the Gene Expression Omnibus (GEO) under accession number GSE169566 (https://www.ncbi.nlm.nih.gov/geo/query/acc.cgi?acc=GSE169566). The remaining data are available within the Article, Supplementary Information or Source Data file.

Field-specific reporting

Please select the one below that is the best fit for your research. If you are not sure, read the appropriate sections before making your selection.

- [x] Life sciences
- [ ] Behavioural & social sciences
- [ ] Ecological, evolutionary & environmental sciences

For a reference copy of the document with all sections, see nature.com/documents/nr-reporting-summary-flat.pdf

Life sciences study design

All studies must disclose on these points even when the disclosure is negative.

Sample size	The samples size was 163 pediatric liver tumors (154 HB and 9 hepatocellular carcinoma) based on data acquired from a cohort study (JPLT-2). Since the incidences of hepatoblastoma and HCC in children are very rare, this study contain the largest cohort for biological analysis in these tumors.
Data exclusions	If the quality and amount of DNA and RNA were insufficient, these samples were excluded. After this exclusion, we analyzed 163 cases.
Replication	Due to the restriction of sample amounts, replicated genetic and epigenetic analysis were difficult to be performed . Some studies such as the mutation analysis of CTNNB1 were done replicated in different sites. Immunohistochemical staining of ASCL2 were also replicated.
Randomization	This study is only retrospective biological study using the samples obtained in the JPLT-2 study. Therefore, randomization was not performed in this study.
Blinding	This study is a retrospective study. Thus, blinding was not applicable.

Reporting for specific materials, systems and methods

We require information from authors about some types of materials, experimental systems and methods used in many studies. Here, indicate whether each material, system or method listed is relevant to your study. If you are not sure if a list item applies to your research, read the appropriate section before selecting a response.

Materials & experimental systems

n/a	Involved in the study	
[ ]	[x]	Antibodies
[ ]	[x]	Eukaryotic cell lines
[x]	[ ]	Palaeontology and archaeology
[x]	[ ]	Animals and other organisms
[ ]	[x]	Human research participants
[ ]	[x]	Clinical data
[x]	[ ]	Dual use research of concern

Methods

n/a	Involved in the study	
[ ]	[x]	ChIP-seq
[x]	[ ]	Flow cytometry
[x]	[ ]	MRI-based neuroimaging

Antibodies

Antibodies used	100X diluted Anti-ASCL2 rabbit polyclonal antibody (Biorbyt Ltd, Cambridge, UK), # orb155740
Validation	For the validation of positive reaction of this Anti-ASCL2 rabbit polyclonal antibody (Biorbyt Ltd, Cambridge, UK), # orb155740 , immunohistochemical staining of human intestinal basal cells was performed.

Eukaryotic cell lines

Policy information about cell lines

Cell line source(s)	HepG2, Cell Resource Center for Biomedical Research at Tohoku University (Sendai, Japan)<br>Huh-6, Japanese Collection of Research Bioresources (Osaka, Japan)
Authentication	Authentication was performed by CRCBR and JCRB.
Mycoplasma contamination	All cell lines tested negative for mycoplasma contamination.
Commonly misidentified lines<br>(See ICLAC register)	No commonly misidentified lines were used.

Human research participants

Policy information about studies involving human research participants

Population characteristics	The tissue samples were obtained form the patients enrolled in JPLT-2 study. The patients characteristic was shown in Supplementary Table 1.
Recruitment	The patients were enrolled in JPLT (Japanese Study Group for Pediatric Liver Tumor) institutions in Japan. (113 centers). All hepatoblastoma and HCC were eligible into this study. However, the children with low performance (< 50% Lansky scale) could not be enroled. The exclusion of these massive aggressive cases might be bias for this study.
Ethics oversight	This study was approved by Hiroshima University Ethics Committee (Hiro-Rin-Hi-No.78).

Note that full information on the approval of the study protocol must also be provided in the manuscript.

Clinical data

Policy information about clinical studies

All manuscripts should comply with the ICMJEguidelines for publication of clinical research and a completedCONSORT checklist must be included with all submissions.

Clinical trial registration	JPLT (Japanese study group for pediatric liver tumor) -2 study (UMIN ID: UMIN000001116).
Study protocol	JPLT2 protocol (CITA and ITEC regimen) for the pediatric liver tumor in Japan. The summary of this protocol is available in the reported paper (J Clin Oncol, 2020: 38: 2488-2498) or JPLT website: https://home.hiroshima-u.ac.jp/eiso/english/home.html.
Data collection	The samples were collected in the patients enrolled between 2000-2012 in JPLT-2 study after signed informed consent was obtained. These patients were followed until 2017 for evaluating primary endpoints.
Outcomes	The outcome of this study was already published (J Clin Oncol, 2020: 38: 2488-2498). In this study, primary endpoints were event-free survival and overall survival (5 years). Secondary endpoints were toxicity, surgical outcome, response of chemotherapy, prognostic factors and molecular analysis.

ChIP-seq

Data deposition

- [x] Confirm that both raw and final processed data have been deposited in a public database such as GEO.
- [x] Confirm that you have deposited or provided access to graph files (e.g. BED files) for the called peaks.

Data access links<br>*May remain private before publication.*	https://www.ncbi.nlm.nih.gov/geo/query/acc.cgi?acc=GSE169566
Files in database submission	GSM5209424: Genome-wide mapping of ASCL2 binding sites in HepG2<br>GSM5209425: Genome-wide mapping of ASCL2 binding sites in Huh-6<br>GSM5209426: Genome-wide mapping of CTNNB1 binding sites in HepG2
Genome browser session<br>(e.g. UCSC)	Genome browser track files (in bigwig format) are available under supplementary files for GSE169566.

Methodology

Replicates	No replicates
Sequencing depth	Sample Total Reads/Pairs Uniquely Aligned Reads/Pairs Experiment Type Seq Type Read Length<br>HepG2_ASCL2 6,188,992 3,240,876 ChIP-Seq Single 50<br>Huh6_ASCL2 4,654,003 2,046,964 ChIP-Seq Single 50<br>HepG2_B_Catinen 30,783,839 20,887,695 ChIP-Seq Single 36

Antibodies	Anti-ASCL2 mouse monoclonal antibody (MERCK, MAB4418, 2930180 and 3430315)<br>Anti-CTNNB1 rabbit polyclonal antibody (Santa Cruz, sc-7963, E0611)
Peak calling parameters	Reads are mapped using bowtie-1.2.1.1 and samtools-0.1.16 with parameters (-S -m 1 -l 36 -n 2).<br>Local coverage is calculated by deepTools package (bamCoverage 2.5.4) with a smoothing length of 300 bp (smoothLength = 300).
Data quality	The number of peaks with standard threshold (>30) and more stringent threshold (>50) is as follows;<br>HepG2_ASCL2 27,718 15,328<br>Huh6_ASCL2 50,656 23,156<br>HepG2_B_Catinen 5,421 1,123
Software	bowtie-1.2.1.1, samtools-0.1.16., deepTools package (bamCoverage 2.5.4), IGV 2.3.98
